# Novel dehydrins lacking complete K-segments in Pinaceae. The exception rather than the rule

**DOI:** 10.3389/fpls.2014.00682

**Published:** 2014-12-02

**Authors:** Pedro Perdiguero, Carmen Collada, Álvaro Soto

**Affiliations:** ^1^GENFOR Grupo de Investigación en Genética y Fisiología Forestal, Universidad Politécnica de MadridMadrid, Spain; ^2^Unidad Mixta de Genómica y Ecofisiología ForestalINIA/UPM, Madrid, Spain

**Keywords:** dehydrins, K-segments, drought, gene expression, qRT-PCR, *Pinus*

## Abstract

Dehydrins are thought to play an essential role in the plant response, acclimation and tolerance to different abiotic stresses, such as cold and drought. These proteins contain conserved and repeated segments in their amino acid sequence, used for their classification. Thus, dehydrins from angiosperms present different repetitions of the segments Y, S, and K, while gymnosperm dehydrins show A, E, S, and K segments. The only fragment present in all the dehydrins described to date is the K-segment. Different works suggest the K-segment is involved in key protective functions during dehydration stress, mainly stabilizing membranes. In this work, we describe for the first time two *Pinus pinaster* proteins with truncated K-segments and a third one completely lacking K-segments, but whose sequence homology leads us to consider them still as dehydrins. qRT-PCR expression analysis show a significant induction of these dehydrins during a severe and prolonged drought stress. By *in silico* analysis we confirmed the presence of these dehydrins in other Pinaceae species, breaking the convention regarding the compulsory presence of K-segments in these proteins. The way of action of these unusual dehydrins remains unrevealed.

## Introduction

Late embryogenesis abundant (LEA) 2 or dehydrin proteins are one of the main components of the response to several abiotic stresses (such as cold or drought) in the Plant kingdom. They constitute a highly complex multigenic family. Dehydrin proteins from angiosperm species are traditionally classified according to the number and order of three highly conserved segments in their amino acid sequence, named Y-, S-, and K-segments (Close, 1996, 1997). On their side, gymnosperm dehydrins are characterized by the absence of Y-segments and, at least in some families, the presence of A- and E-segments (Perdiguero et al., 2012a).

The only segment present in every dehydrin described to date is the K-segment. In angiosperms it consists in a highly conserved lysine rich 15-mer, with a consensus sequence EKKGIMDKIKEKLPG (Close, 1996), while in gymnosperms it shows a more variable sequence: (Q/E)K(P/A)G(M/L)LDKIK(A/Q)(K/M)(I/L)PG (Jarvis et al., 1996).

Although the biological role of the highly conserved K-segment is not yet established it is thought to play an essential role during abiotic stress responses (Close, 1996; Svensson et al., 2002). It may be involved in conformational changes through the formation of class A2 amphipathic α-helix. This helix is supposed to establish hydrophobic interactions with other proteins, stabilizing cell membranes (Campbell and Close, 1997; Danyluk et al., 1998; Koag et al., 2003). A study with *dhn1* from maize concluded that the K-segment is necessary and sufficient for binding to anionic phospholipid vesicles (Koag et al., 2009). Also, the K-segments of *dhn5* from wheat have been described as essential for the protection of two enzymes, lactate dehydrogenase and β-glycosidase (Drira et al., 2013). Similar results were reported for *dhn5* from *Rhododendron catawbiense* and ERD10 from *Arabidopsis*, in which a deleterious effect on protective capacity of lactate dehydrogenase were observed when K-segments of these dehydrins were total or partially removed (Reyes et al., 2008). Antibacterial activity has also been reported for *A. thaliana* ERD10: a deletion study showed that K-segments are responsible of *in vivo* inhibition on *E. coli* cells (Campos et al., 2006). This effect was later validated using synthetic K-segments from a rice dehydrin which showed *in vitro* antibacterial activity, particularly against Gram-positive bacteria (Zhai et al., 2011).

Some peculiarities have been reported for three unusual dehydrins from citrus, COR11, COR15, and COR19, responsive to low temperature. These proteins differ from most other plant dehydrins by having a K-segment similar to that of gymnosperms and by having a serine cluster (S-segment) at an unusual position at the carboxy-terminus (Cai et al., 1995; Porat et al., 2002; Talon and Gmitter, 2008).

Here we report the identification and structural characterization of three novel dehydrin genes in *Pinus pinaster* which are characterized by the absence of a complete K-segment in the amino acid sequence. We have also confirmed by *in silico* analysis the presence of this unusual dehydrin in other conifer species. Additionally, we report that transcription of these genes is inducible by dehydration, as confirmed by quantitative RT-PCR analysis of their expression patterns in different organs during a severe and prolonged drought stress.

## Materials and methods

### Plant material and treatment conditions

The plant material and drought treatment described in Perdiguero et al. (2012a) was used for this study. *P. pinaster* clonal material of three different genotypes (F1P3, F2P2, F4P4) from Oria provenance (37° 30′ 30″ N 2° 20′ 20″ W, southeastern Spain) was grown in containers with peat:perlite:vermiculite (3:1:1). One year old cuttings were kept in growth chambers for 2 months with a photoperiod of 16/8 (day/night), with a temperature of 24°C and 60% of relative humidity during the day and 20°C and 80% of relative humidity during the night, and watered at field capacity prior to drought treatment.

Four ramets per genotype were collected at each sampling point. Unstressed plants were harvested 1 h after the last watering. The remaining plants were maintained without irrigation and collected every 10 days (five sampling points, S1–S5). Needles, stem and roots from each plant were collected separately, immediately frozen in liquid nitrogen and stored at −80°C.

### Sequence analysis

Tentative contigs (TCs) assembled from ESTs corresponding to putative dehydrins of pines were searched in the Pine Gene Index 9.0 (http://compbio.dfci.harvard.edu/tgi/; release March 2011). TCs corresponding to unusual putative dehydrins from *Pinus sp* never reported in the literature were selected and used as query in SustainPineDB (version 3.0), a database containing the *de novo* assembled transcriptome from *Pinus pinaster* (Canales et al., 2013). Unigenes obtained this way were used to manually design primers (following Innis and Gelfand, 1990, recommendations) flanking the complete ORF for further PCR amplification from both gDNA and cDNA.

Identification of putative orthologous sequences was performed using BLASTP and TBLASTN software in GenBank databases as well as in High Confidence Genes database version 1.0 of Norway spruce genome project, available in ConGenIE website. BioEdit was used to transcribe nucleotide sequences to amino acid sequences and MUSCLE software (Edgar, 2004) was used to align deduced amino acid sequences. Maximum likelihood methods were applied to estimate phylogeny of *Pinus pinaster* dehydrins using the software PhyML 3.0 (Guindon et al., 2010). Both alignment and phylogeny analysis were performed in the website Phylogeny.fr (Dereeper et al., 2008). DISOPRED3 (Ward et al., 2004) and Phyre2 (Kelley and Sternberg, 2009) softwares were used to identify the putative secondary structure and disordered regions.

### DNA and RNA isolation and gene searching

Genomic DNA was extracted from needles and megagametophytes following Doyle (1990), with slight modifications. Total RNA was isolated separately from roots, stem and needles following a CTAB–LiCl precipitation method (Chang et al., 1993). cDNA was synthesized from 1μg of total RNA using PowerScriptIII reverse transcriptase (Invitrogen). Complete sequences for each studied dehydrin were amplified by PCR, using cDNA and genomic DNA as templates and specific primers (Table 1). The PCR products were cloned into pGEM®T-easy vector (Promega, WI, USA) and transformed into *Escherichia coli* DH5α cells. The obtained clones were sequenced and aligned using Spidey mRNA-to-genomic software (http://www.ncbi.nlm.nih.gov/spidey/) to reveal the exon-intron structure of the genes.

**Table 1:** **Specific primers used in isolation of complete ORF and RT-PCR**.

**Dehydrin**	**Forward**	**Sequence (5 ′-3 ′)**	**Reverse**	**Sequence (5 ′-3 ′)**	**Length genomic**	**Length cDNA**
**Specific primers used in isolation of complete ORF**						
*Ppter_dhn_SK′a*	SK′a_FW	ATATTTGAATTTGCAGGTTGATAACT	SK′a_RV	CGCTCCTCCTTCCGTTTCTA	708 bp	545 bp
*Ppter_dhn_SK′b*	SK′b_FW	GGTTGATAGCTTTTCAAATTACC	SK′b_RV	CTTCCGTTACCATGGACTTC	665 bp	522 bp
*Ppter_dhn_S*	S_FW	GAATTTGCAGGTTGATAGCTT	S_RV	GGATCTTCCTGCTGTTACTTA	687 bp	544 bp
**Specific primers used in RT-PCR**					**Length amplicon**	
*Ppter_dhn_SK′a*	SK′a_RT_FW	AAGGAGAAAATGCACGTTGG	SK′a_RT_RV	GCTGGATGATGATAAGGTGC	89 bp	
*Ppter_dhn_SK′b*	SK′b_RT _FW	GGCAGGAAAAAGGAAGAAAGGA	SK′b_RT _RV	TGCAGCAGCAGCAGCTAGATA	120 bp	
*Ppter_dhn_S*	S_RT _FW	CGGCAAGAATAAGGACGGAAAT	S_RT _RV	GCGGAGCAGCCACAGCTA	122 bp	
*Ri18S*	Ri18S_RT_FW	GCGAAAGCATTTGCCAAGG	Ri18S_RT_RV	ATTCCTGGTCGGCATCGTTTA	110 bp	

### Real-time quantitative PCR

Total RNA from roots, stem and needles of each plant was treated with DNAse Turbo (Ambion; Applied Biosystems, Life Technologies, CA, USA). First-strand cDNA was synthesized from 2μg of total RNA from each sample using PowerScriptIII reverse transcriptase (Invitrogen, Life Technologies, Paisley, UK) according to the supplier's manual. 18S rRNA was used as a control, after verifying that the signal intensity remained unchanged across all treatments. Primer Express v. 3.0.0 (Applied Biosystems Life Technologies, CA, USA) software was used to design PCR primers. Amplified fragments were sequenced to check reaction specificity, and primers were modified when needed in order to avoid cross amplification. Final primers are shown in Table 1. Polymerase chain reactions were performed in an optical 96-well plate with a CFX 96 Detection system (BIO-RAD), using EvaGreen to monitor dsDNA synthesis. Reactions containing 2x SsoFast EvaGreen Supermix reagent (BIO-RAD, CA, USA), 12.5 ng cDNA and 500 nM of primers in a final volume of 10μl were subjected to the specific thermal profile. Three technical replicates were performed for each PCR run. The expression ratios were then obtained using the ΔΔCT method corrected for the PCR efficiency for each gene (Pfaffl, 2001).

### Sequences deposition

The sequences obtained in this study were submitted to the GenBank with the following accessions numbers; KM033833–KM033835 for mRNA and KM033843–KM033845 for genomic DNA.

## Results

### *In silico* identification of K-segment lacking dehydrins

**Figure 1:**
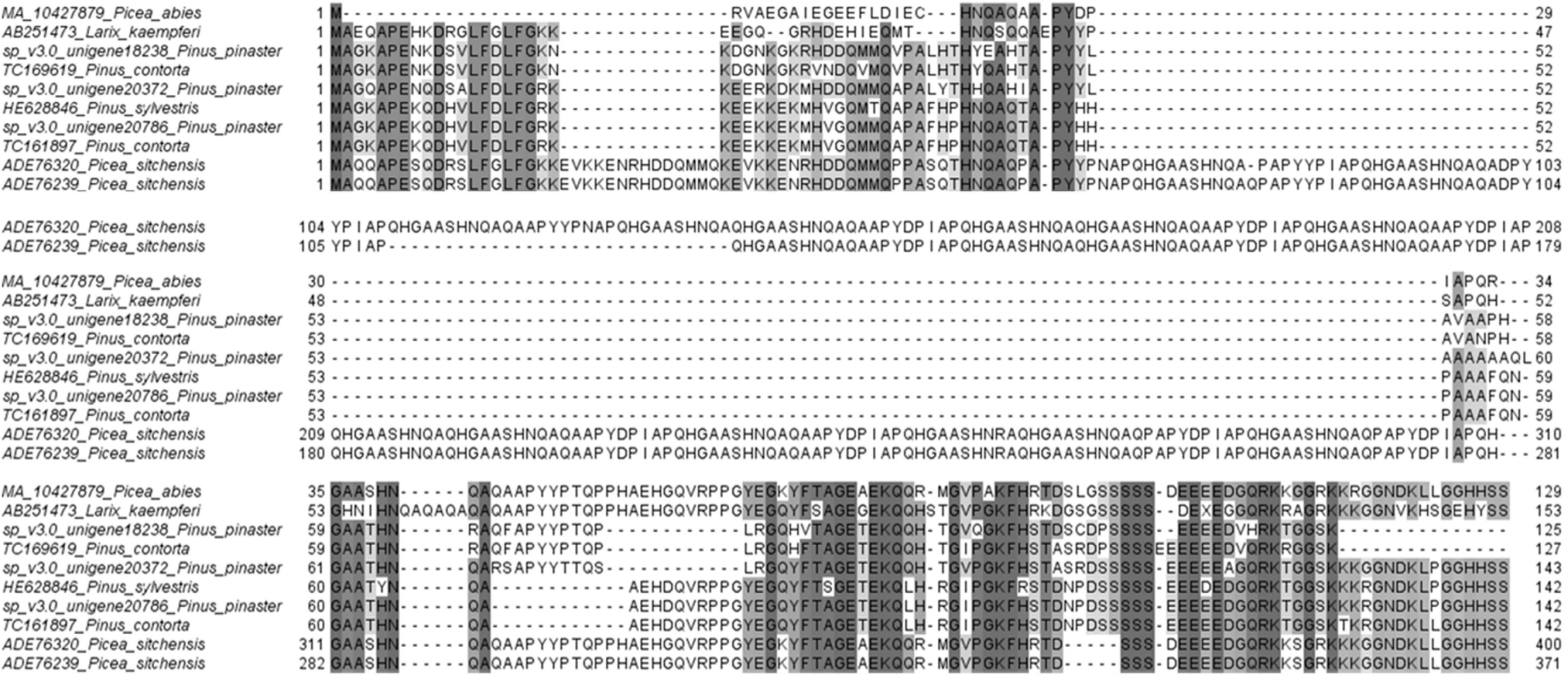
**Optimized alignment performed with MUSCLE software of dehydrins from conifer species identified in different databases**. All dehydrins share the absence of complete K-segments in their amino acid sequences.

Exhaustive search of dehydrin sequences in the Pine Gene Index allowed the identification of 47 full amino acid sequences in a previous work (Perdiguero et al., 2012a). Analysis of the sequences considered incomplete then, led to the identification of two TCs from *Pinus contorta* (TC169619 and TC161897), which do not present a complete K-segment. Blast searching in SustainPineDB using these sequences as query resulted in three unigenes from *P. pinaster* that encoded putative full dehydrins (sp_v3.0_unigene20786, sp_v3.0_unigene18238 and sp_v3.0_unigene20372). Other homologous were found in different conifer species (such as *Pinus sylvestris, Picea abies, Picea sitchensis* or *Larix kaempferi*) by searching in Genbank databases. Figure 1 shows an alignment of these amino acid sequences.

### Isolation and analysis of K-segment lacking dehydrins from *pinus pinaster*

**Figure 2:**
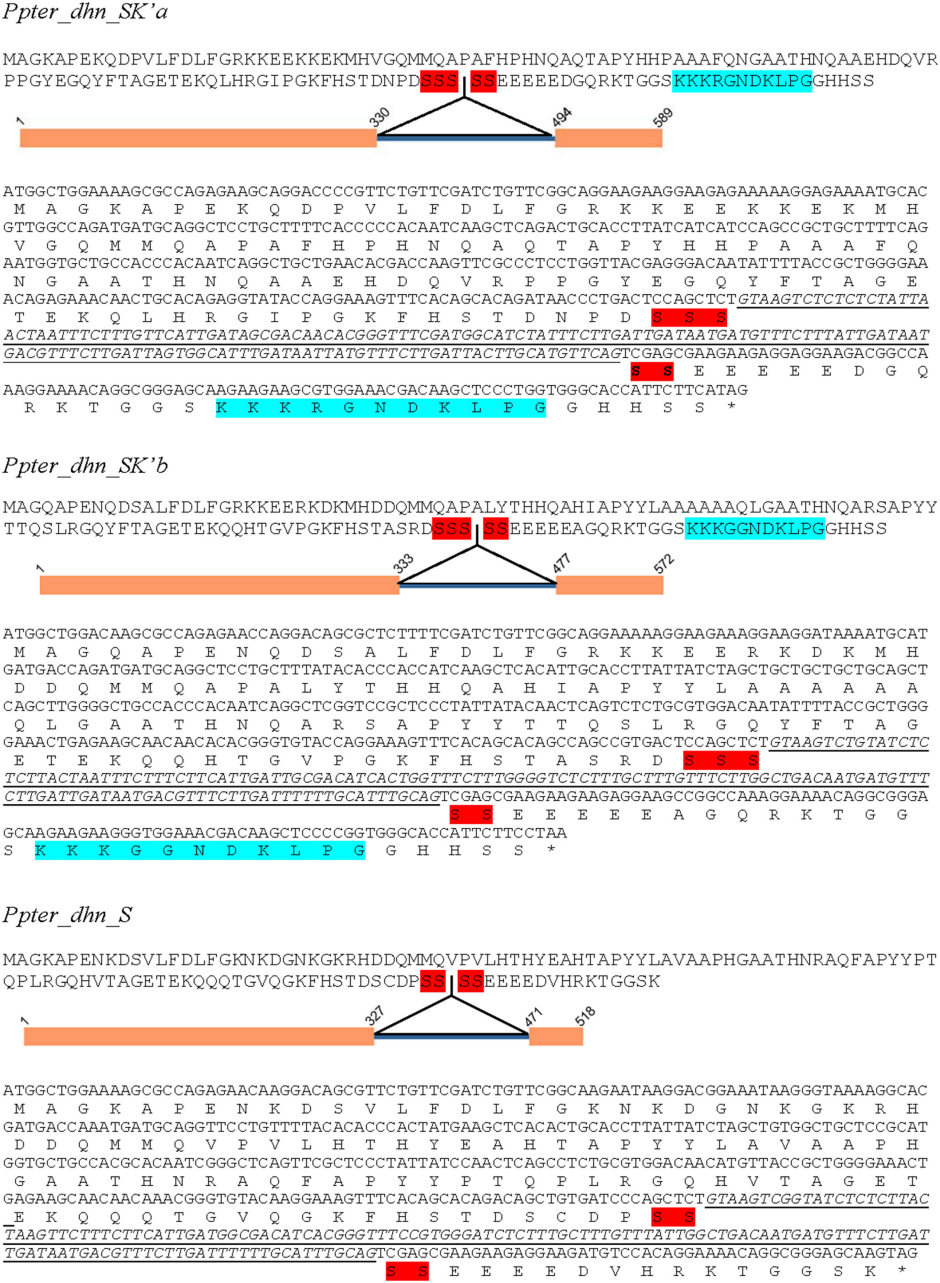
**Nucleotide and amino acid sequences of *Ppter_dhn_SK'a, Ppter_dhn_SK'b*, and *Ppter_dhn_S***. A schematic figure shows the exon-intron structure. Conserved amino acid segments are highlighted. Red, S-segments; Blue, partial K-segments.

*Pinus pinaster* sequences were used to design specific PCR primers (Table 1). PCR amplification from genomic DNA and cDNA from water stressed *P. pinaster* plants have led to the isolation of three full ORF (Supplementary Figure S1). Sequencing of haploid genomic DNA from megagametophytes confirmed their presence at three different loci within the *P. pinaster* genome. They were named as *Ppter_dhn_SK'a* (sp_v3.0_unigene20786) *Ppter_dhn_SK'b* (sp_v3.0_unigene20372) and *Ppter_dhn_S* (sp_v3.0_unigene18238), according to the conserved segments present in their amino acid sequences (Figure 2). All of them show a very short S-segment and two of them also have a modified and truncated K-segment (K').

*Ppter_dhn_SK'a* has a 426 nucleotide-long ORF encoding a protein with 142 amino acids, and pI 7.20. This sequence accumulates several modifications in the region corresponding to the A-segment, showing the sequence Q**A**QT**A**P**Y**H (in bold the conserved residues, compared with the consensus sequence EAASYYP). It has a very short S-segment composed by 5 serine residues. Comparison of the genomic and cDNA sequences allowed the identification of a 163 nucleotide-long intron within the S-segment. It also presents a modified K-segment with 11 amino acids (**KKK**RGN**D—-KLPG**; in bold the conserved residues). On its side, the deduced amino acid sequence of *Ppter_dhn_SK'b* is formed by 143 amino acids, with pI 8.00. It presents two putative modified A-segments (Q**A**HI**A**P**YY**L and Q**A**RS**A**P**YY**T). This sequence also presents a 5 serine-long S-segment with an intron of 143 nucleotides in it and a similar modified K-segment (**KKK**GGN**D**—-**KLPG**). Finally, *Ppter_dhn_S* has an ORF with 375 nucleotides, encoding a 125 amino acids long protein, with pI 6.76. The two putative A-segments present several modifications, as in the other two sequences (**EA**HT**A**P**YY**L and R**A**QF**A**P**YYP**). It shows a very short S-segment, with only 4 serine residues, and a 143 nucleotide-long intron in it. No K-segment can be detected in this sequence.

High percentages of disordered regions, ranging from 64 to 77%, were predicted for the three sequences; several fragments were identified as potential region for α-helix whereas few places were identified to produce β-strand (Supplementary Figure S2).

**Figure 3:**
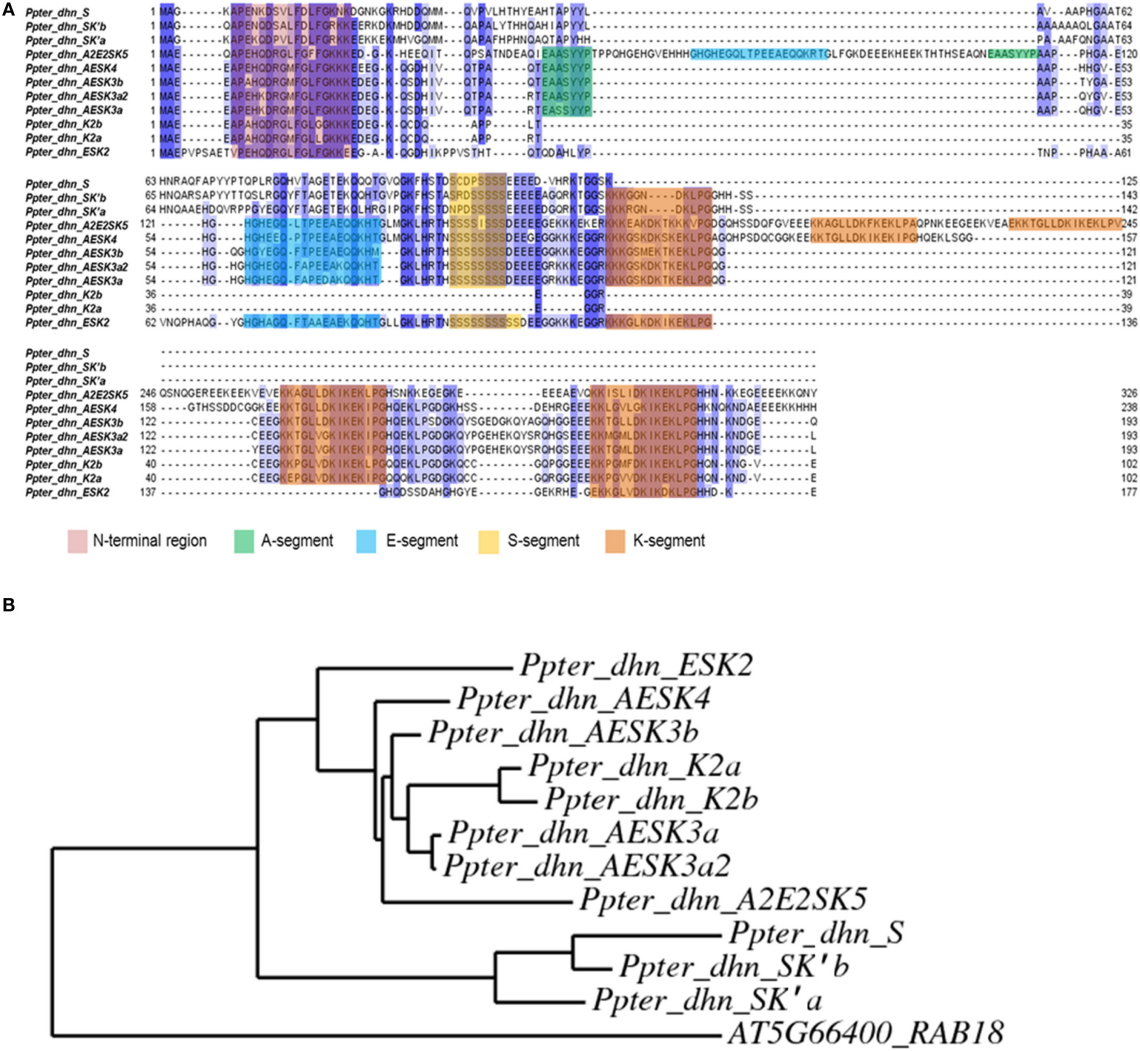
**(A)** Alignment (performed with MUSCLE software) of amino acid sequences corresponding to *Pinus pinaster* dehydrins from Perdiguero et al. (2012a) and dehydrins isolated in the present work. Conserved segments (N-terminal, A, E, S, and K) are highlighted. **(B)** Phylogenetic tree (performed with PhyML software) of *Pinus pinaster* dehydrins. AT5G66400_RAB18 from *Arabidopsis thaliana* has been used as outgroup.

The alignment of the amino acid sequences of these three novel dehydrins with the eight dehydrins described previously in *P. pinaster* and their phylogenetic relationships are shown in Figure 3.

### Expression of *dhn-S* from *pinus pinaster*

Quantitative RT-PCR analysis of the expression patterns of *Ppter_dhn_SK'a, Ppter_dhn_SK'b*, and *Ppter_dhn_S* during a severe and prolonged drought stress were carried out independently in roots, stems and needles of three genotypes from Oria. This provenance, in southeastern Spain, has previously been shown to have a good inducible response to water stress (Sánchez-Gómez et al., 2010) and has been used for the selection of candidate genes involved in the response to water deficit (Perdiguero et al., 2012b).

**Figure 4:**
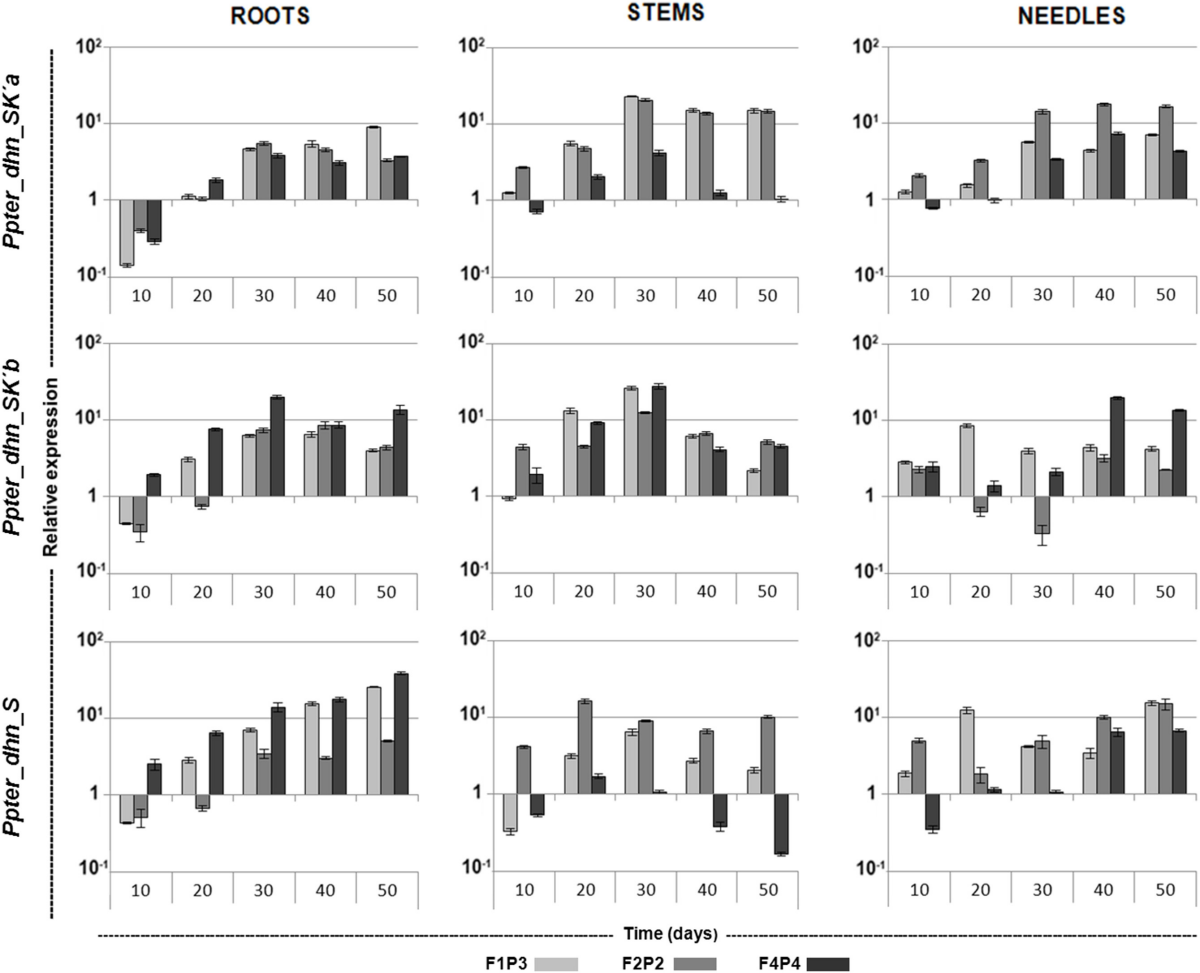
**qRT-PCR expression profiles of *Ppter_dhn_SK'a, Ppter_dhn_SK'b*, and *Ppter_dhn_S* in roots, stems and needles of three different genotypes along a drought stress treatment, relative to unstressed (control) plants**. Three technical replicates per genotype (F1P3, F2P2, and F4P4) were used at each point. Standard errors are shown.

Noticeable increases in transcription level have been detected for the three dehydrins during the drought stress (Figure 4). Thus, transcription levels of *Ppter_dhn_SK'a* increased during the first steps of the experiment. Maximum inductions were detected after 30 days without watering, when transcription levels in roots, leaves and stems of stressed plants reached average values 5-, 8-, and 16-fold higher, respectively, than in unstressed plants. After that, transcription levels were roughly maintained in roots and needles, and decreased slowly in stems. On its side, transcription of *Ppter_dhn_SK'b* in roots also increased with drought stress, reaching its maximum value at 30–40 days (approximately 8-fold higher compared with unstressed plants, and even 20-fold higher for one of the genotypes). Transcription induction was faster and stronger in stems, reaching maximum values at 30 days (more than 20-fold higher levels) and decreasing afterwards. On the contrary, the pattern was less consistent in needles, with noticeable variability among genotypes. Finally, *Ppter_dhn_S* showed a continuously increasing transcription in roots throughout the experiment, reaching values more than 25-fold higher in two of the genotypes after 50 days without watering. Maximum values were also attained at the end of the experiment in needles, while the pattern was less consistent in stems, with maximum inductions at 20–30 days. Afterwards, transcription decreased, reaching values even 6-fold lower than in control plants at the fifth sampling point for one of the genotypes, while the others still showed values 2- and 10-fold higher than in control plants.

## Discussion

In this work we identified and characterized three novel proteins from *Pinus pinaster* whose overall homology leads us to consider them as dehydrins. However, they present several noticeable differences with usual dehydrins. For instance, they show several modifications in the N-terminal region (Figure 3), rather well conserved among Pinaceae dehydrins (Perdiguero et al., 2012a). They also accumulate discrepancies in the A-segment and present extremely short S-segments, with 4–5 serine residues, while other *P. pinaster* dehydrins have 7–9 residues. The three genes include an intron within the S-segment, a feature also found in other *P. pinaster* dehydrins (Perdiguero et al., 2012a), as well as in SKn-type dehydrins from angiosperms (Jiménez-Bremont et al., 2013).

However, the most remarkable difference appears in the short C-terminal region (starting from the S-segment), with the absence of a proper K-segment. Close (1996) provided a detailed description of dehydrins, analyzing the complete sequences available at the moment (67 from angiosperm and 3 from gymnosperm). He concluded that the dehydrin family is unified by the presence of one or more copies of the K-segment. This characteristic has been confirmed in all dehydrins described in plants up to date, including several studies analyzing complete genomes. For instance, the dehydrin protein family has been analyzed in the moss *Phycomitrella patens* (Ruibal et al., 2012), in herbaceous species as *Arabidopsis thaliana* (Bies-Ethève et al., 2008; Hundertmark and Hincha, 2008) or *Oryza sativa* (Wang et al., 2007), in different legumes (Battaglia and Covarrubias, 2013) and tree species as *Prunus mume* (Du et al., 2013) or *Populus trichocarpa* (Liu et al., 2012; Lan et al., 2013). All the dehydrins identified in these works present K-segments in their amino acid sequences, although with different modifications in certain cases. The information available is so robust that the presence of at least one K-segment have been assumed as necessary in recent reviews (Kosová et al., 2010; Eriksson and Harryson, 2011; Hanin et al., 2011), while other homologous genomic sequences lacking K-segment have been annotated as uncharacterized or hypothetical proteins and not as dehydrins (f.i., XP_008240561 from *Prunus mume*, XP_007201808.1 from *Prunus persica* or XP_004234737.1 from *Solanum lycopersicon*).

Strikingly, two of the proteins described here present shortened and modified K-segments, which we have called K'-segments, while the third one absolutely lacks this region. However, these three proteins show high overall homology levels with typical dehydrins, especially in certain regions (N-terminal region, S-segment and modified K-segment; Figure 3), which leads us to consider them still as dehydrins. They also share structural characteristics with usual dehydrins. For instance, regarding their predicted secondary structure, several putative α-helix are identified, but a high proportion (70%) of their sequences, especially in the less conserved regions, is classified as disordered. These percentages are comparable to the ones predicted for other dehydrins, not only in *P. pinaster* but also in angiosperm species as *Populus trichocarpa* or *Arabidopsis thaliana* (Supplementary Figure S2). It is believed that this feature, the high percentage of disordered secondary structure, is relevant to the fulfillment of the protective role of dehydrins during water stress (Mouillon et al., 2008; Hughes and Graether, 2011).

Additionally, the three genes described here have shown a noticeable increase in transcription levels under drought stress. This induction is similar or even higher than those reported for other “orthodox,” K and AESK dehydrins in *P. pinaster* (Perdiguero et al., 2012a). In general, transcription increases throughout the experiment, reaching a maximum at 30–50 days without watering. Expression patterns are more consistent in roots, which could be related to the key role played by this organ in detecting and triggering the response to water stress. On the contrary, more variability is detected in the expression patterns in stems and leaves. In all the cases, the overall transcription levels are low and, certainly, induction is not comparable to that of the drought-responsive *Ppter_dhn_ESK_2_* (Perdiguero et al., 2012a). However, as reported for other dehydrins, we cannot discard that these K-segment lacking dehydrins are effective in protection against drought stress even at low concentrations, and/or that they could be involved in other processes different from drought stress.

Consistently, *in silico* analysis of available RNA-seq data from different libraries of *Picea abies* (Nystedt et al., 2013) shows also differential expression of MA_10427879g0010, the putative orthologous gene of the ones reported here. For instance, noticeable transcription induction is detected in needles at midday, as well as in stems and needles of girdled twigs, in which hydraulic conductivity is affected and dehydration processes are registered (Supplementary Figure S3).

Pines have abundant repetitions and pseudogenes in their huge genomes (Morse et al., 2009). At a first glance these loci might seem an example of this sort of repetitions, which eventually could, in the course of evolution, further diverge or even become pseudogenes. Two of the sequences reported here present a degenerated K-segment and the third one completely lacks this segment, which is considered to be relevant for dehydrin functionality. Additionally, high variability in their transcription under water stress has been observed among genotypes and organs, which could be seen as an indirect evidence of the no functionality of these three proteins. Nevertheless, maybe the 8 conserved residues in the K-segments of two of them (**KKK**X**G**X-**D—-KLPG**) could be determinant for α-helix formation and protective activity. Further *in vivo* and *in vitro* experiments are needed to clarify the effect of these modifications and to confirm if these proteins are actually functional or not.

## Author contributions

Pedro Perdiguero performed the laboratory work. Pedro Perdiguero and Álvaro Soto drafted the manuscript. Carmen Collada and Álvaro Soto conceived and designed the experiments. All authors contributed to writing the article and approved the final manuscript.

### Conflict of interest statement

The authors declare that the research was conducted in the absence of any commercial or financial relationships that could be construed as a potential conflict of interest.
