# Supplementary material for: Novel dehydrins lacking complete K-segments in Pinaceae. The exception rather than the rule
Source: Front Plant Sci. 2014 Dec 2;5:682. doi: 10.3389/fpls.2014.00682 (PMC4251312; doi:10.3389/fpls.2014.00682)
Supplement: Supplementary file 1 [file Image1.PDF]

Novel dehydrins lacking complete K-segments in Pinaceae. The exception rather than the rule

Pedro Perdiguero, Carmen Collada, Álvaro Soto

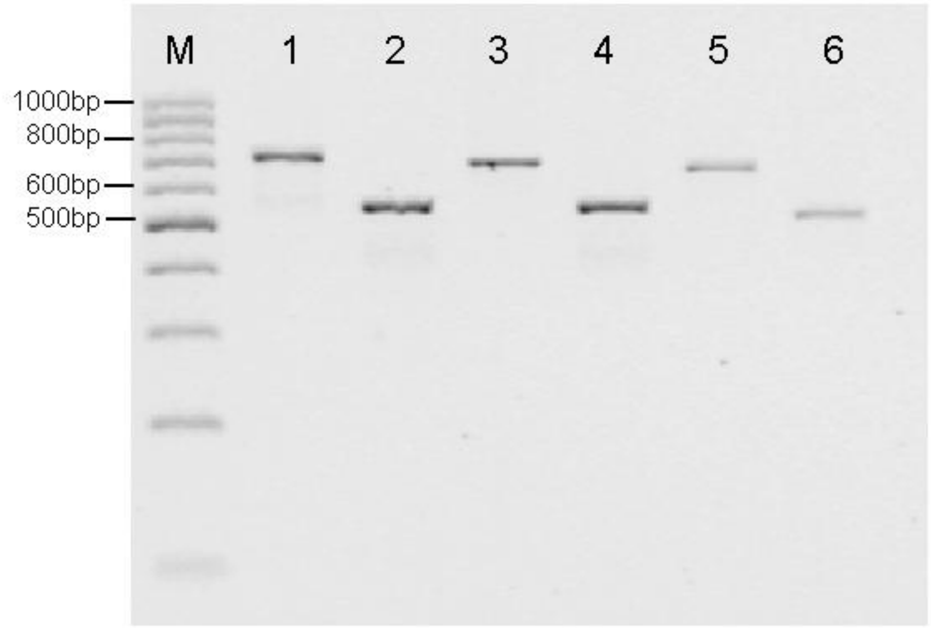

Suppl. Fig. S1 PCR products corresponding to *Ppter_dhn_SK'a* (lanes 1 and 2), *Ppter_dhn_S* (3, 4) and *Ppter_dhn_SK'b* (5, 6) loci amplified from haploid genomic DNA isolated from megagametophytes (1, 3, 5) and cDNA (2, 4, 6). M: DNA Ladder
