# Supplementary material for: Novel dehydrins lacking complete K-segments in Pinaceae. The exception rather than the rule
Source: Front Plant Sci. 2014 Dec 2;5:682. doi: 10.3389/fpls.2014.00682 (PMC4251312; doi:10.3389/fpls.2014.00682)
Supplement: Supplementary file 2 [file Image2.PDF]

Novel dehydrins lacking complete K-segments in Pinaceae. The exception rather than the rule

Pedro Perdiguero, Carmen Collada, Álvaro Soto

Suppl. Fig. S2; **A)** Disordered regions predicted with **DISOPRED3** software for the new proteins identified in this work and **B)** Disordered regions and secondary structure predicted with **Phyre 2** software for the new proteins as well as the dehydrins previously described from *P. pinaster* (Perdiguero *et al.* 2012a) and all the dehydrins identified in *Arabidopsis thaliana* (Hundertmark *et al*. 2008) and *Populus trichocarpa* (Liu *et al*. 2012 and Lan *et al*. 2013) genomes.

A. Disordered regions in the amino acid sequence of *Ppter_dhn_SK'a*, *Ppter_dhn_SK'b* and *Ppter_dhn_S* as predicted by **DISOPRED3** software. Blue lines represent the confidence score of disorder prediction. Regions are considered disordered when this score is higher than 0.5 (dashed red lines). Orange lines represent the confidence score of the prediction of protein binding sites within the disordered regions.

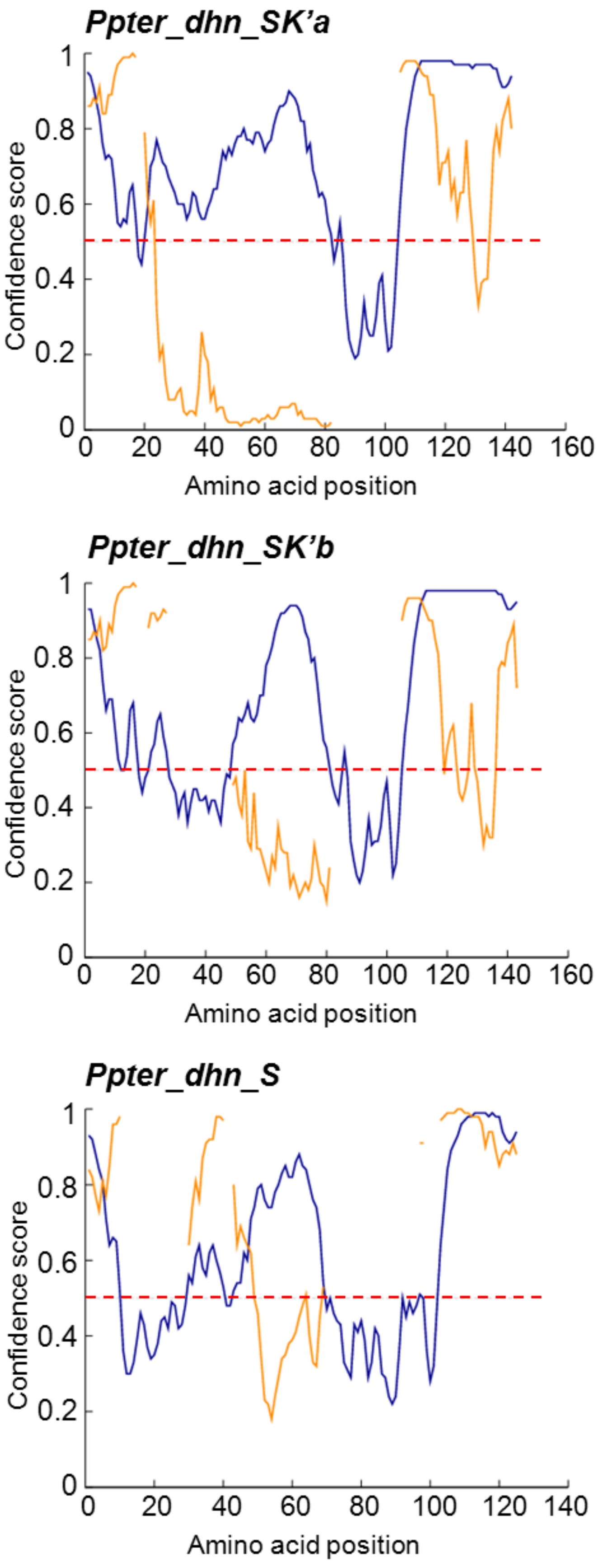

B. Disordered regions and secondary structure predicted with **Phyre 2** software. In 'Secondary structure' line green helices represent α-helices, blue arrows indicate β-strands and faint lines indicate coil. The 'Disorder' line contains the prediction of disordered regions in your protein and such regions are indicated by question marks (?). The 'SS or Disorder confidence' line indicates the confidence in the prediction, with red being high confidence and blue low confidence.

Index

1. *Ppter_dhn_SK'a*
2. *Ppter_dhn_SK'b*
3. *Ppter_dhn_S*
4. *Ppter_dhn_K2a*
5. *Ppter_dhn_K2b*
6. *Ppter_dhn_ESK2*
7. *Ppter_dhn_AESK3a*
8. *Ppter_dhn_AESK3a2*
9. *Ppter_dhn_AESK3b*
10. *Ppter_dhn_AESK4*
11. *Ppter_dhn_A2E2SK5*
12. *AT1G20440*
13. *AT1G20450*
14. *AT1G54410*
15. *AT1G76180*
16. *AT2G21490*
17. *AT3G50970*
18. *AT3G50980*
19. *AT4G38410*
20. *AT4G39130*
21. *AT5G66400*
22. *Potri_002G013200*
23. *Potri_003G138700*
24. *Potri_004G158500*
25. *Potri_005G248100*
26. *Potri_009G120100*
27. *Potri_013G062100*
28. *Potri_013G062200*
29. *Potri_013G062300*
30. *Potri_013G062400*

Email	pedro.perdiguero@upm.es
Description	Ppter_dhn_SK_a
Date	Mon Sep 15 14:47:26 BST 2014
Unique Job ID	595925c7499cbab3

Secondary structure and disorder prediction

1 . . . . 10 . . . . 20 . . . . 30 . . . . 40 . . . . 50 60

Sequence: M A G K A P E K Q D P V L F D L F G R K K E E K K E K M H V G Q M M Q A P A F H P H N Q A Q T A P Y H H P A A A F Q N G

Secondary structure

SS confidence

Disorder: ?

Disorder confidence

. . . . 70 . . . . 80 . . . . 90 . . . . 100 . . . . 110 120

Sequence: A A T H N Q A A E H D Q V R P P G Y E G Q Y F T A G E T E K Q L H R G I P G K F H S T D N P D S S S S S E E E E E D G Q

Secondary structure

SS confidence

Disorder: ?

Disorder confidence

. . . . 130 . . . . 140 . .

Sequence: R K T G G S K K K R G N D K L P G G H H S S

Secondary structure

SS confidence

Disorder: ?

Disorder confidence

Confidence Key

High(9) Low (0)

? Disordered (77%)

Alpha helix (37%)

Beta strand (2%)

Phyre²

Email	pedro.perdiguero@upm.es
Description	Ppter_dhn_SK_b
Date	Mon Sep 15 14:46:50 BST 2014
Unique Job ID	15d13c0808c1cac3

Secondary structure and disorder prediction

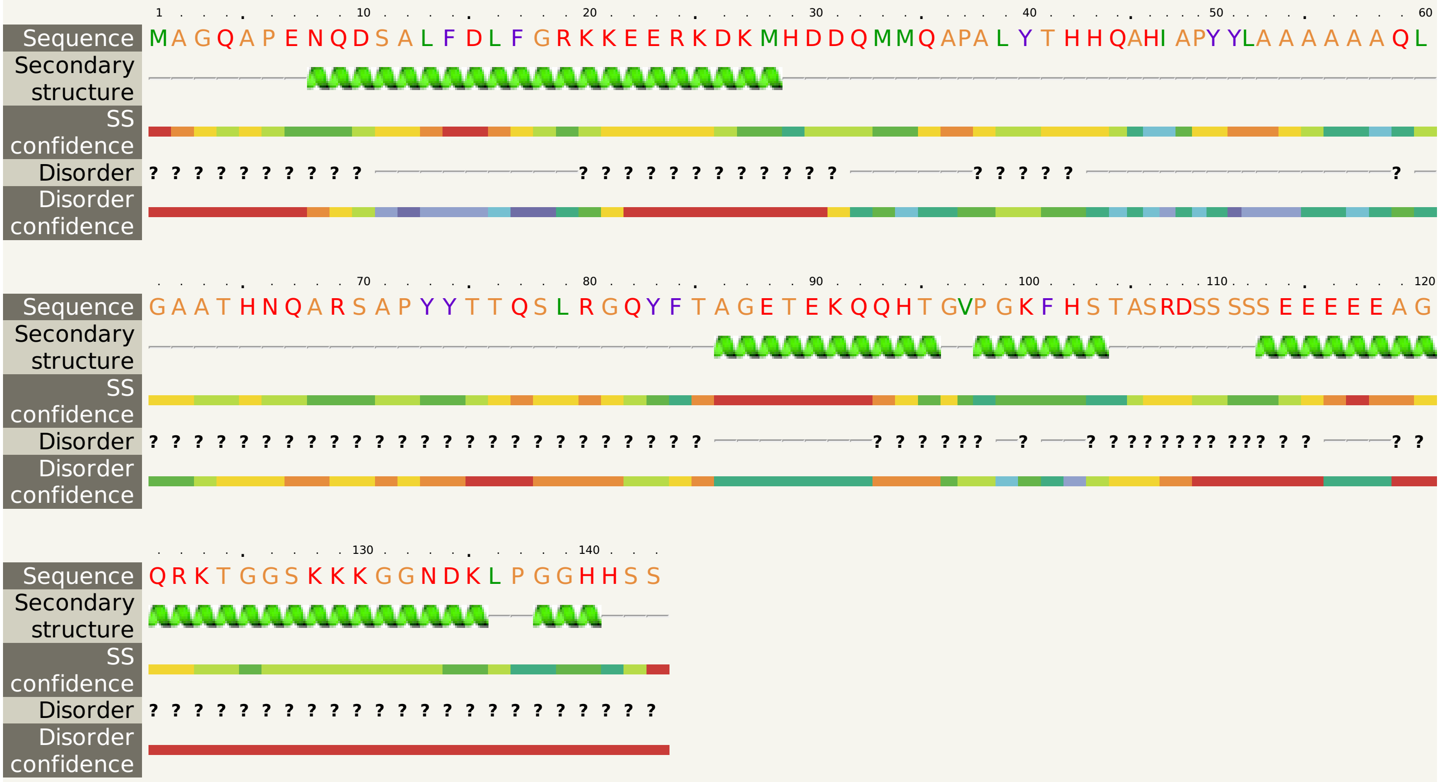

Confidence Key

High(9) Low (0)

? Disordered (69%)

Alpha helix (44%)

Beta strand (0%)

Phyre^2

Email	pedro.perdiguero@upm.es
Description	Ppter_dhn_S
Date	Mon Sep 15 14:47:54 BST 2014
Unique Job ID	9620d2d9f254a674

Secondary structure and disorder prediction

1 . . . . 10 . . . . 20 . . . . 30 . . . . 40 . . . . 50 60

Sequence M A G K A P E N K D S V L F D L F G K N K D G N K G K R H D D Q M M Q V P V L H T H Y E A H T A P Y Y L A V A A P H G A

Secondary structure

SS confidence

Disorder ? ???????? ? ? ???? ? ? ? ? ? ? ? ? ? ? ? ???? ?? ? ? ? ?

Disorder confidence

. . . . 70 . . . . 80 . . . . 90 . . . . 100 . . . . 110 120

Sequence A T H N R A Q F A P Y Y P T Q P L R G Q H V T A G E T E K Q Q Q T G V Q G K F H S T D S C D P S S S S E E E E D V H R K

Secondary structure

SS confidence

Disorder ? ???????? ? ? ? ? ? ? ? ? ? ????? ? ? ? ? ? ? ????????? ? ? ? ?

Disorder confidence

. . . . .

Sequence T G G S K

Secondary structure

SS confidence

Disorder ? ????

Disorder confidence

Confidence Key

High(9) Low (0)

? Disordered (64%)

Alpha helix (34%)

Beta strand (2%)

Email	pedro.perdiguero@upm.es
Description	Ppter_dhn_K2a
Date	Mon Sep 15 14:42:20 BST 2014
Unique Job ID	0b606a006e07d7db

Phyre2

Secondary structure and disorder prediction

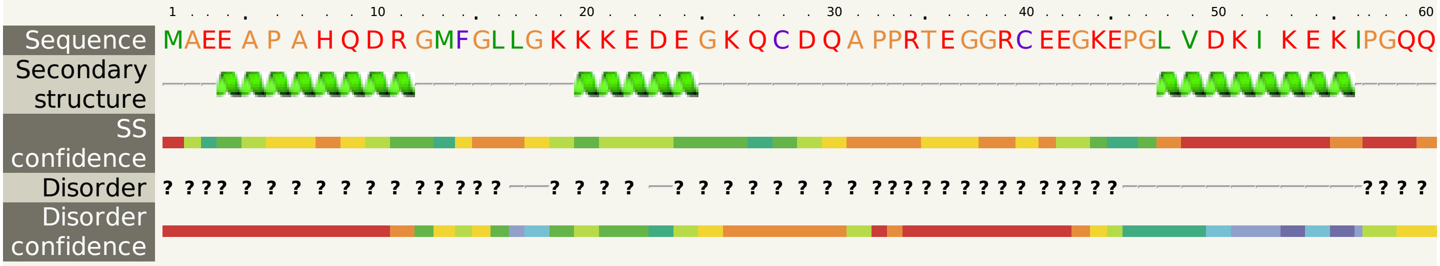

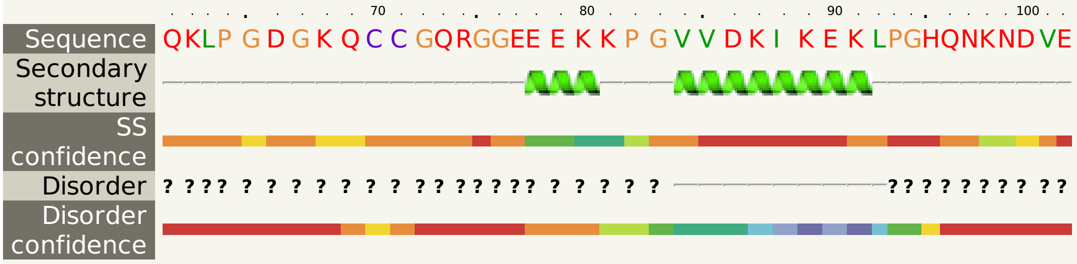

Confidence Key

High(9) Low (0)

? Disordered (77%)

Alpha helix (31%)

Beta strand (0%)

Email	pedro.perdiguero@upm.es
Description	Ppter_dhn_K2b
Date	Mon Sep 15 14:43:02 BST 2014
Unique Job ID	67ebc7dc3dae5ac9

Secondary structure and disorder prediction

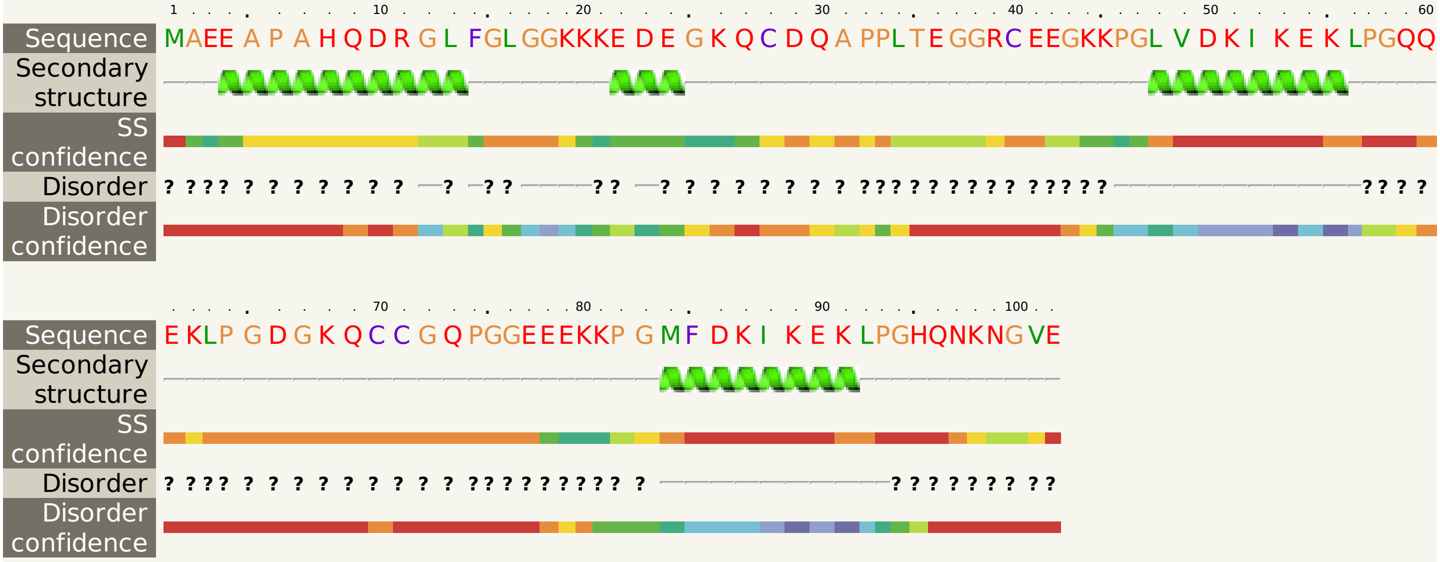

Confidence Key

High(9) Low (0)

? Disordered (73%)

Alpha helix (28%)

Beta strand (0%)

Field	Value
Email	pedro.perdiguero@upm.es
Description	Ppter_dhn_ESK2
Date	Mon Sep 15 14:43:32 BST 2014
Unique Job ID	9d8ec3ddbda19782

Secondary structure and disorder prediction

Sequence (1–60): M A E P V P S A E T V P E H Q D R G L F G L F G K K E E G A K Q G D H I K P P V S T H T Q T Q D A H L Y P T N P P H A A

Sequence (61–120): A V N Q P H A Q G Y G H G H A G Q F T A A E A E K Q Q H T G L L G K L H R T N S S S S S S S S S S D E E G G K K K E G G

Sequence (121–177): R K K K G L K D K I K E K L P G G H Q D S S D A H G H G Y E G E K R H E G E K K G L V D K I K D K L P G H H D K E

Confidence Key

High(9) … Low (0)

? Disordered (77%)

Alpha helix (27%)

Beta strand (0%)

Phyre2

Email	pedro.perdiguero@upm.es
Description	Ppter_dhn_AESK3a
Date	Mon Sep 15 14:43:52 BST 2014
Unique Job ID	0a9b70ecb6f009be

Secondary structure and disorder prediction

Position	Sequence
1–60	M A E E A P E H K D R G M F G L F G K K K E D E G K Q S D H I V Q T P A R T E A S S Y Y P A A P Q H G V E H G H G H G H
61–120	E G Q F A P E D A K Q Q K H T G K L H R T H S S S S S S S S D E E E E G R K K K E G G R K K K G S K D K T K E K L P G Q
121–180	G Y E E G K K T G L V G K I K E K I P G H Q E K L P G D G K Q Y P G E H E K Q Y S R Q H G S E E E K K T G M L D K I K E
181–193	K L P G H H N K N D G E L

Rows per block: Sequence; Secondary structure; SS confidence; Disorder; Disorder confidence

Confidence Key

High(9) … Low (0)

? Disordered (67%)

Alpha helix (54%)

Beta strand (1%)

Email	pedro.perdiguero@upm.es
Description	Ppter_dhn_AESK3a2
Date	Mon Sep 15 14:44:15 BST 2014
Unique Job ID	6f8d66e363f4d464

Secondary structure and disorder prediction

Sequence (1–60): MAEEAPEHKDRGMFGLFGKKKEDEGKQSDHIVQTPARTEAASYYPAAPQYGVEHGHGHGH

Sequence (61–120): EGQFAPEEAKQQKHTGKLHRTHSSSSSSSSDEEEEGRKKKEGGRKKKGSKDKTKEKLPGQ

Sequence (121–180): GCEEGKKTGLVGKIKEKIPGHQEKLPGDGKQYPGEHEKQYSRQHGSEEEKKMGMLDKIKE

Sequence (181–193): KLPGHHNKNDGEL

Rows: Sequence, Secondary structure, SS confidence, Disorder, Disorder confidence

Confidence Key

High(9) … Low (0)

? Disordered (76%)

Alpha helix (53%)

Beta strand (0%)

Phyre2

Field	Value
Email	pedro.perdiguero@upm.es
Description	Ppter_dhn_AESK3b
Date	Mon Sep 15 14:44:36 BST 2014
Unique Job ID	cc0812fa61f5d973

Secondary structure and disorder prediction

1 . . . . 10 . . . . 20 . . . . 30 . . . . 40 . . . . 50 60

Sequence: MAEEAPAHQDRGMFGLFGKKKEDEGKQSDHIVQTPAQTEAASYYPAAPTYGAEHGQGHGY

Secondary structure

SS confidence

Disorder

Disorder confidence

. . . . 70 . . . . 80 . . . . 90 . . . . 100 . . . . 110 120

Sequence: EGQFTPEEAEQQKHMGKLHRTHSSSSSSSSDEEEEGGKKKEGGRKKKGSMEKTKEKLPGQ

Secondary structure

SS confidence

Disorder

Disorder confidence

. . . . 130 . . . . 140 . . . . 150 . . . . 160 . . . . 170 180

Sequence: GCEEGKKTGLLDKIKEKIPGHQEKLPSDGKQYSGEDGKQYAGQHGGEEEKKTGLLDKIKE

Secondary structure

SS confidence

Disorder

Disorder confidence

. . . . 190 . . .

Sequence: KLPGHHNKNDGEQ

Secondary structure

SS confidence

Disorder

Disorder confidence

Confidence Key

High(9) — Low (0)

? Disordered (78%)

Alpha helix (49%)

Beta strand (0%)

Phyre2

Email	pedro.perdiguero@upm.es
Description	Ppter_dhn_AESK4
Date	Mon Sep 15 14:45:03 BST 2014
Unique Job ID	9aad27730639ad9e

Secondary structure and disorder prediction

1 … 10 … 20 … 30 … 40 … 50 … 60

Sequence: M A E E A P E H Q D R G M F G L F G K K K E E E G K Q G D H I V Q T P A Q T E A A S Y Y P A A P H H G V E H G H G H E E

70 … 80 … 90 … 100 … 110 … 120

Sequence: Q P T P E E A E Q Q K H T G L M G K L H R T N S S S S S S S S D E E G E G G K K K E G G R K K K G S K D K S K E K L P G

130 … 140 … 150 … 160 … 170 … 180

Sequence: A G Q H P S D Q C G G K E E K K T G L L D K I K E K I P G H Q E K L S G G G T H S S D D C G G K E E K K T G L L D K I K

190 … 200 … 210 … 220 … 230

Sequence: E K I P G H Q E K L P G D G K H S S D E H R G E E E K K L G V L G K I K E K L P G H K N Q K N D A E E E E K K H H H

Confidence Key

High(9) Low (0)

? Disordered (92%)

Alpha helix (38%)

Beta strand (0%)

Phyre²

Email	pedro.perdiguero@upm.es
Description	Ppter_dhn_A2E2SK5
Date	Mon Sep 15 14:45:28 BST 2014
Unique Job ID	55f8730f9d46c35f

Secondary structure and disorder prediction

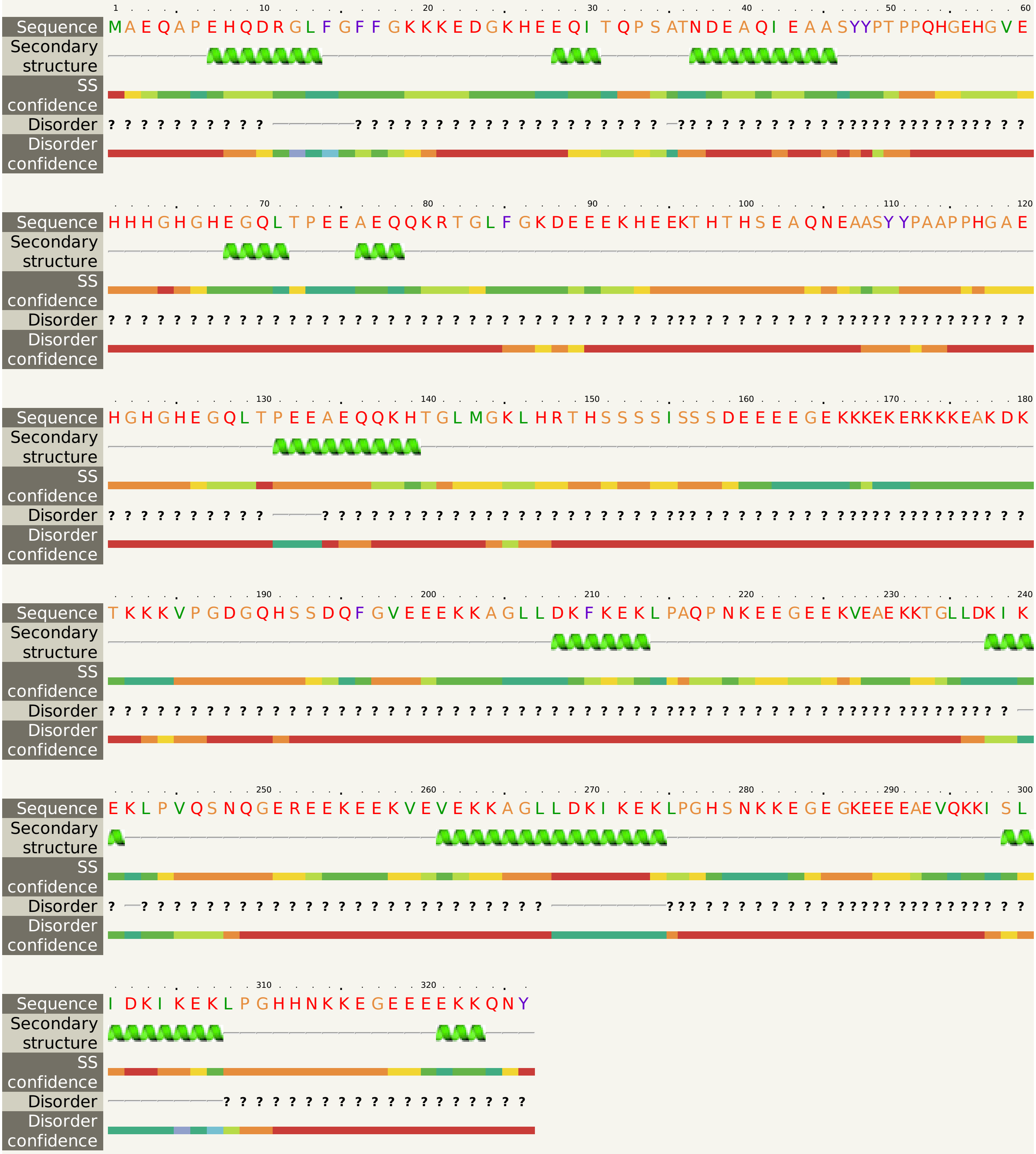

Confidence Key

High(9) Low (0)

? Disordered (92%)

Alpha helix (22%)
Beta strand (0%)

Email	pedro.perdiguero@upm.es
Description	Arabidopsis_thaliana__AT1G20440
Date	Mon Sep 15 15:14:23 BST 2014
Unique Job ID	dafe055c3a894212

Secondary structure and disorder prediction

Sequence 1–60: M A E E Y K N N V P E H E T P T V A T E E S P A T T T E V T D R G L F D F L G K K E E E V K P Q E T T T L E S E F D H K

Sequence 61–120: A Q I S E P E L A A E H E E V K E N K I T L L E E L Q E K T E E D E E N K P S V I E K L H R S N S S S S S S S D E E G E

Sequence 121–180: E K K E K K K K I V E G E E D K K G L V E K I K E K L P G H H D K T A E D D V P V S T T I P V P V S E S V V E H D H P E

Sequence 181–240: E E K K G L V E K I K E K L P G H H D E K A E D S P A V T S T P L V V T E H P V E P T T E L P V E H P E E K K G I L E K

Sequence 241–: I K E K L P G Y H A K T T E E E V K K E K E S D D

Confidence Key

High(9) … Low (0)

? Disordered (83%)

Alpha helix (29%)

Beta strand (1%)

Email	pedro.perdiguero@upm.es
Description	Arabidopsis_thaliana__AT1G20450
Date	Mon Sep 15 15:14:46 BST 2014
Unique Job ID	9636034cd62c42c9

Phyre2

Secondary structure and disorder prediction

	1–60
Sequence	MAEEYKNTVPEQETPKVATEESSAPEIKERGMFDFLKKKEEVKPQETTTLASEFEHKTQI
Secondary structure	
SS confidence	
Disorder	
Disorder confidence	

	61–120
Sequence	SEPESFVAKHEEEEHKPTLLEQLHQKHEEEEENKPSLLDKLHRSNSSSSSSSDEEGEDGE
Secondary structure	
SS confidence	
Disorder	
Disorder confidence	

	121–180
Sequence	KKKKEKKKKIVEGDHVKTVEEENQGVMDRIKEKFPLGEKPGGDDVPVVTTMPAPHSVEDH
Secondary structure	
SS confidence	
Disorder	
Disorder confidence	

	181–240
Sequence	KPEEEEKKGFMDKIKEKLPGHSKKPEDSQVVNTTPLVETATPIADIPEEKKGFMDKIKEK
Secondary structure	
SS confidence	
Disorder	
Disorder confidence	

	241–260
Sequence	LPGYHAKTTGEEEKKEKVSD
Secondary structure	
SS confidence	
Disorder	
Disorder confidence	

Confidence Key

High(9) Low (0)

? Disordered (89%)

Alpha helix (13%)

Beta strand (0%)

Email	pedro.perdiguero@upm.es
Description	Arabidopsis_thaliana__AT1G54410
Date	Mon Sep 15 15:15:07 BST 2014
Unique Job ID	9517946a37587d4c

Secondary structure and disorder prediction

1 . . . . 10 . . . . 20 . . . . 30 . . . . 40 . . . . 50 60

Sequence: MAGLINKIGDALHIGGGNKEGEHKKEEEHKKHVDEHKSGEHKEGIVDKIKDKIHGGEGKS

Secondary structure

SS confidence

Disorder: ?

Disorder confidence

. . . . 70 . . . . 80 . . . . 90 . . . .

Sequence: HDGEGKSHDGEKKKKKDKKEKKHHDDGHHSSSSDSDSD

Secondary structure

SS confidence

Disorder: ?

Disorder confidence

Confidence Key

High(9) Low (0)

? Disordered (69%)

Alpha helix (61%)

Beta strand (0%)

Email	pedro.perdiguero@upm.es
Description	Arabidopsis_thaliana__AT1G76180
Date	Mon Sep 15 15:13:49 BST 2014
Unique Job ID	676ea4a9f1e6890d

Secondary structure and disorder prediction

Sequence (1–60): M A E E I K N V P E Q E V P K V A T E E S S A E V T D R G L F D F L G K K K D E T K P E E T P I A S E F E Q K V H I S E

Sequence (61–120): P E P E V K H E S L L E K L H R S D S S S S S S S E E E G S D G E K R K K K K E K K K P T T E V E V K E E E K K G F M E

Sequence (121–180): K L K E K L P G H K K P E D G S A V A A A P V V V P P P V E E A H P V E K K G I L E K I K E K L P G Y H P K T T V E E E

Sequence (181–185): K K D K E

Confidence Key

High(9) … Low (0)

? Disordered (82%)

Alpha helix (26%)

Beta strand (0%)

Email	pedro.perdiguero@upm.es
Description	Arabidopsis_thaliana__AT2G21490
Date	Mon Sep 15 15:13:24 BST 2014
Unique Job ID	bd45e0fbb8569cd0

Secondary structure and disorder prediction

	1 . . . . 10 . . . . 20 . . . . 30 . . . . 40 . . . . 50 60
Sequence	MADLRDEKGNPIHLTDTQGNPIVDLTDEHGNPMYLTGVVSSTPQHKESTTSDIAEHPTST
Secondary structure	
SS confidence	
Disorder	? ?
Disorder confidence	

	. . . . 70 . . . . 80 . . . . 90 . . . . 100 . . . . 110 120
Sequence	VGETHPAAAPAGAGAATAATATGVSAGTGATTTGQQHHGSLEEHLRRSGSSSSSSSEDDG
Secondary structure	
SS confidence	
Disorder	? ?
Disorder confidence	

	. . . . 130 . . . . 140 . . . . 150 . . . . 160 . . . . 170 180
Sequence	QGGRRKKSIKEKIKEKFGSGKHKDEQTPATATTTGPATTDQPHEKKGILEKIKDKLPGHH
Secondary structure	
SS confidence	
Disorder	? ?
Disorder confidence	

	. . . . .
Sequence	NHNHP
Secondary structure	
SS confidence	
Disorder	? ? ? ? ?
Disorder confidence	

Confidence Key

High(9) Low (0)

? Disordered (78%)

Alpha helix (12%)

Beta strand (2%)

Email	pedro.perdiguero@upm.es
Description	Arabidopsis_thaliana__AT3G50970
Date	Mon Sep 15 15:16:24 BST 2014
Unique Job ID	832fff13c592a37a

Secondary structure and disorder prediction

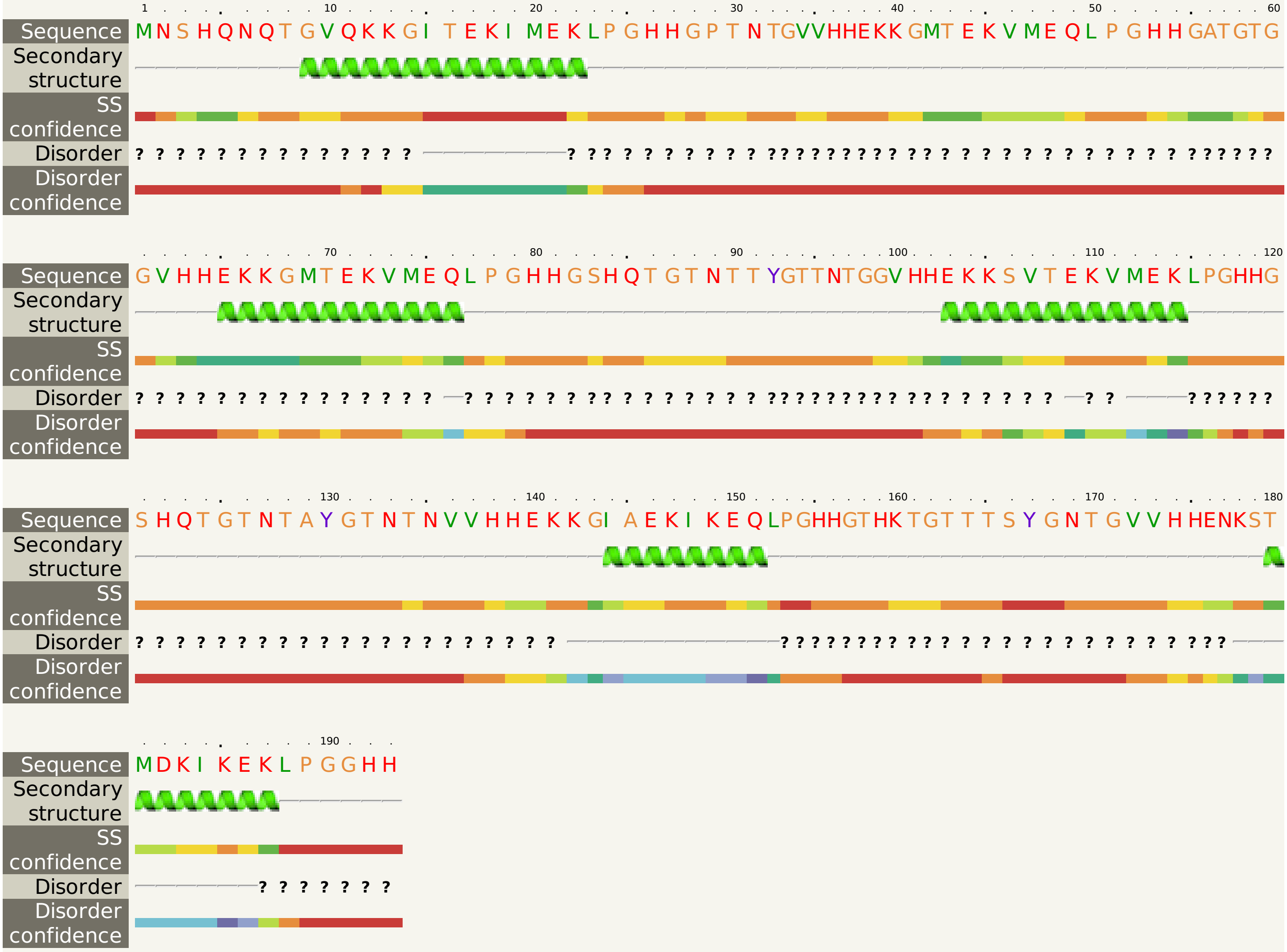

Confidence Key

High(9) Low (0)

? Disordered (83%)

Alpha helix (28%)

Beta strand (0%)

Email	pedro.perdiguero@upm.es
Description	Arabidopsis_thaliana__AT3G50980
Date	Mon Sep 15 15:16:01 BST 2014
Unique Job ID	3f70595b474a8b95

Secondary structure and disorder prediction

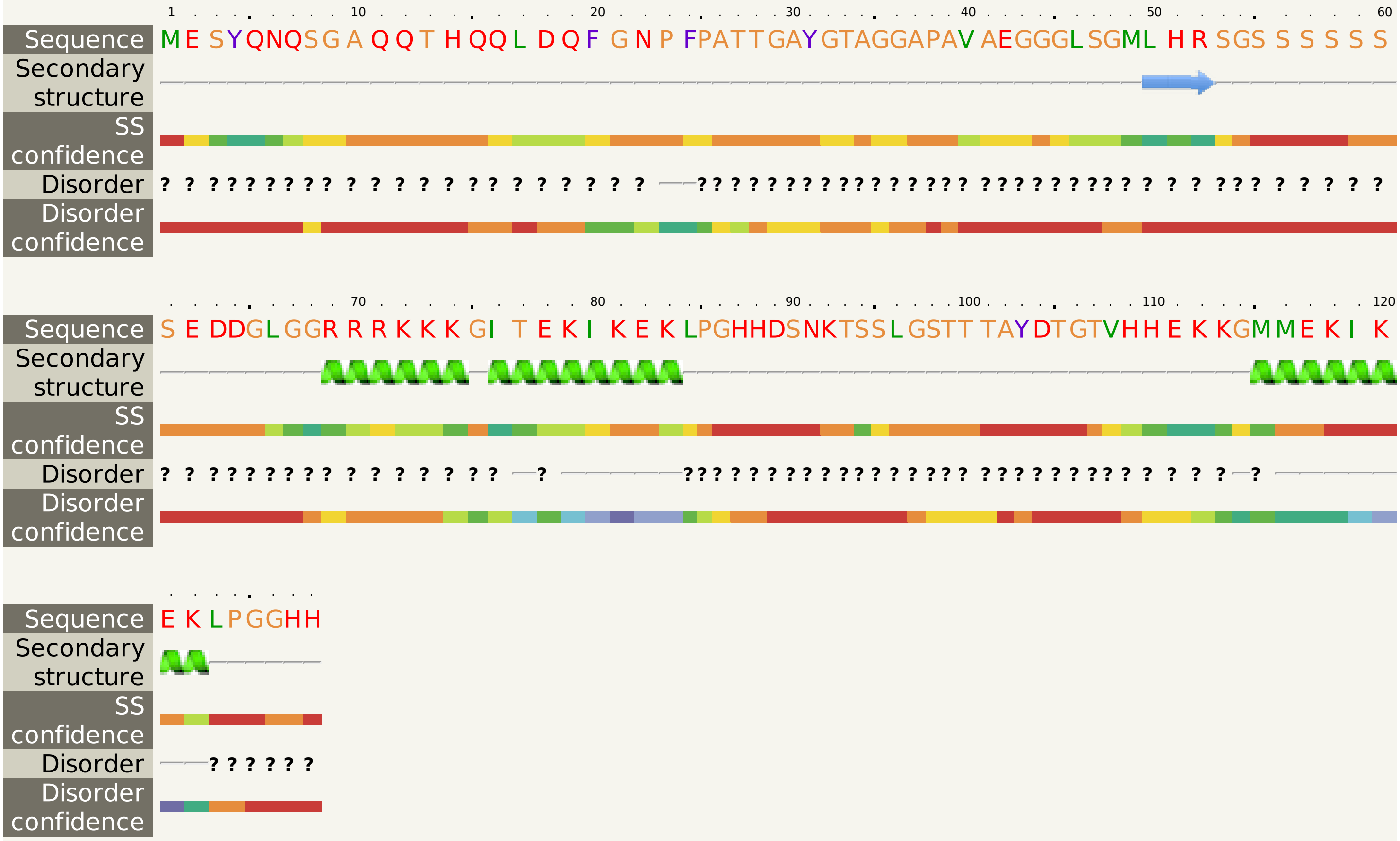

Confidence Key

High(9) Low (0)

? Disordered (88%)

Alpha helix (17%)

Beta strand (2%)

Email	pedro.perdiguero@upm.es
Description	Arabidopsis_thaliana__AT4G38410
Date	Mon Sep 15 15:12:45 BST 2014
Unique Job ID	c3488d442448b63c

Secondary structure and disorder prediction

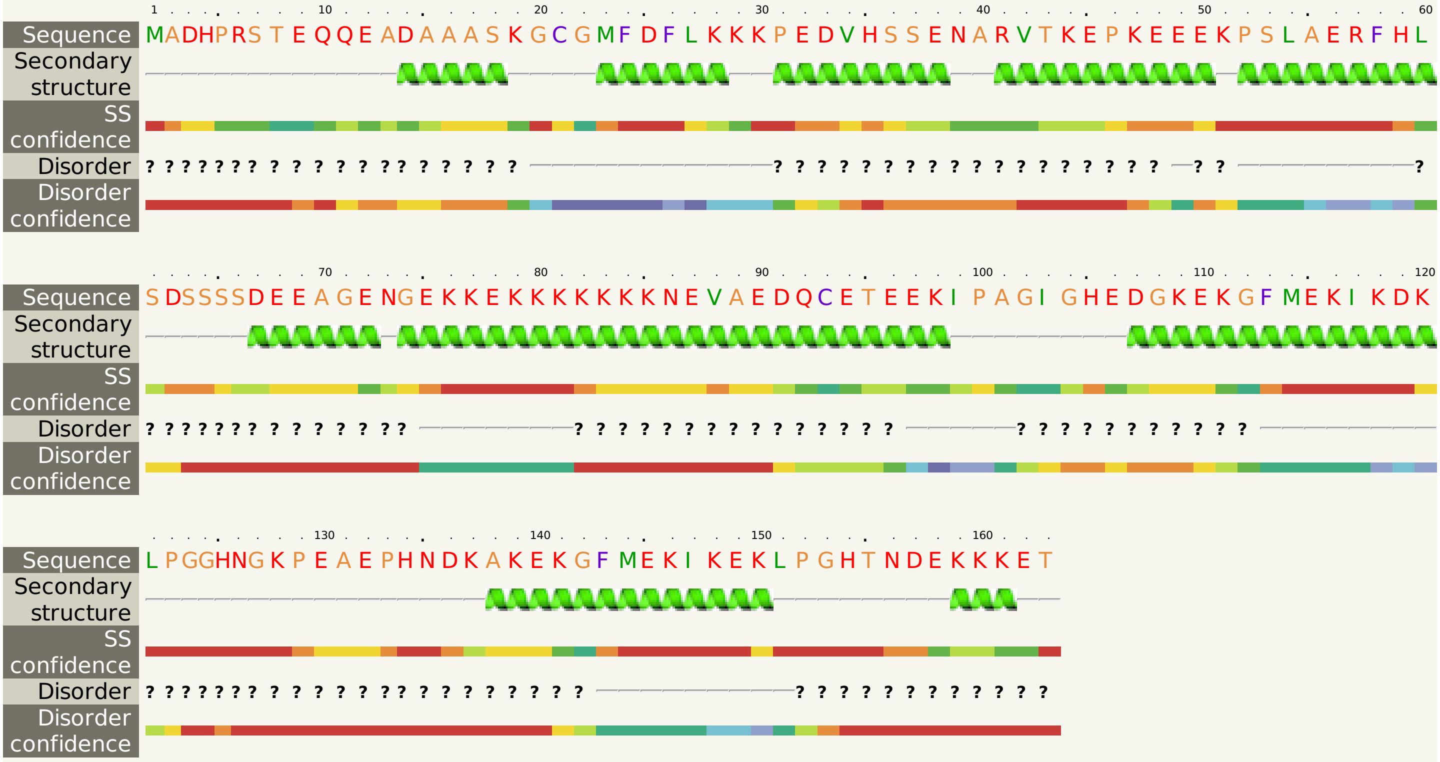

Confidence Key

High(9) Low (0)

? Disordered (70%)

Alpha helix (61%)

Beta strand (0%)

Email	pedro.perdiguero@upm.es
Description	Arabidopsis_thaliana__AT4G39130
Date	Mon Sep 15 15:13:05 BST 2014
Unique Job ID	4737b2f08a4483b0

Phyre2

Secondary structure and disorder prediction

	1 … 60
Sequence	MADLKDERGNPIYLTDAHGEPAQLMDEFGNAMHLTGVATTVPHLKESSYTGPHPITAPVT
Secondary structure	
SS confidence	
Disorder	??????? ? ? ? ? ? ?? ?? ?????? ? ?????? ? ? ? ?????????
Disorder confidence	

	61 … 120
Sequence	TTNTPHHAQPISVSHDPLQDHDLRWFGTSSTEENGEGVGRKTNITDETKSKLGVDKPSAA
Secondary structure	
SS confidence	
Disorder	?????????? ? ? ? ? ? ? ? ? ? ? ? ? ? ?? ?????? ? ? ??????? ? ?????? ? ? ? ? ?????????
Disorder confidence	

	121 … 151
Sequence	TVTGSGSGSVHEKKGFFKKIKEKLSGHHNDL
Secondary structure	
SS confidence	
Disorder	?????????? ? ? ? ?? ?????? ?
Disorder confidence	

Confidence Key

High(9) Low (0)

? Disordered (81%)

Alpha helix (17%)

Beta strand (7%)

Phyre2

Email	pedro.perdiguero@upm.es
Description	Arabidopsis_thaliana__AT5G66400
Date	Mon Sep 15 15:15:35 BST 2014
Unique Job ID	8d9c1ed359f79405

Secondary structure and disorder prediction

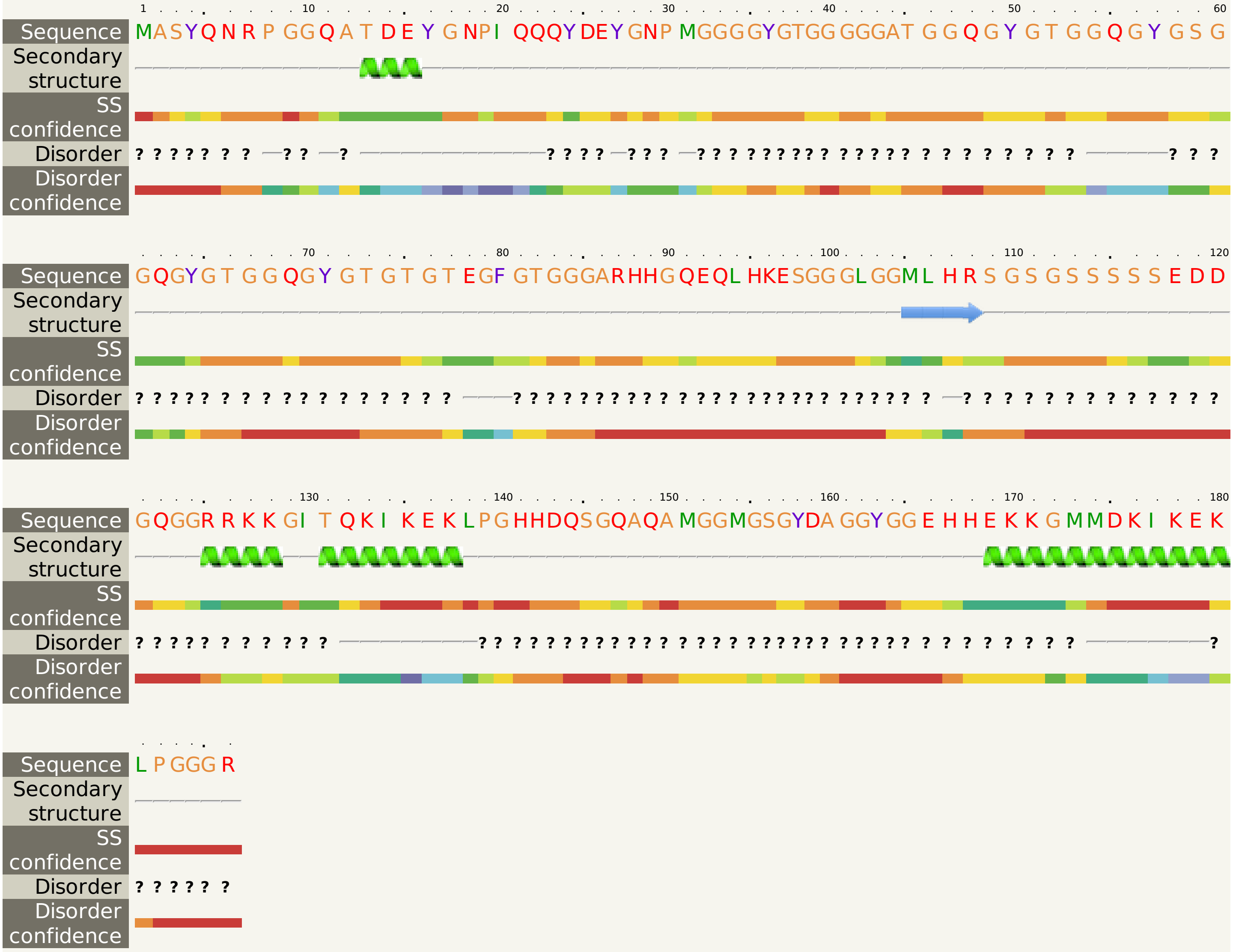

Confidence Key

High(9) Low (0)

? Disordered (81%)

Alpha helix (14%)

Beta strand (2%)

Email	pedro.perdiguero@upm.es
Description	Populus_trichocarpa_Potri_002G013200
Date	Mon Sep 15 15:20:48 BST 2014
Unique Job ID	de74a39b6c32dfc3

Secondary structure and disorder prediction

1 . . . . 10 . . . . 20 . . . . 30 . . . . 40 . . . . 50 60

Sequence: M A G V N K S H E Y E T K T S A G E E S G A T E T K D Q T P L T P E K K K S Y F E Q A K D M I P A Y K K T E E V P P P T

Secondary structure

SS confidence

Disorder: ? ? ? ? ? ? ? ? ? — — ?

Disorder confidence

. . . . 70 . . . . 80 . . . . 90 . . . . 100 . . . . 110 120

Sequence: E A V Y P S E T P L T P E K K K S Y F E Q A K G I I P A Y K K T E D G P P S P A E A A V H P T G T P L E P E K K K S Y F

Secondary structure

SS confidence

Disorder: ?

Disorder confidence

. . . . 130 . . . . 140 . . . . 150 . . . . 160 . . . . 170 180

Sequence: E Q A K D M I P A Y K K T E D S P P S P A E A A V H P T G S R P L E P E K K S Y F E Q A K D M I P A Y K K T E D S P P S

Secondary structure

SS confidence

Disorder: ?

Disorder confidence

. . . . 190 . . . . 200 . . . . 210 . . . . 220 . . . . 230 240

Sequence: P A E A A V H P T G S R P L E P E K K K S Y F E Q A K D I I P A Y K K T E D S P P S P T E A A A H P T G S R P L E P E K

Secondary structure

SS confidence

Disorder: ?

Disorder confidence

. . . . 250 . . . . 260 . . . . 270 . . . . 280 . . . . 290 300

Sequence: K K S Y F E Q A K E R T P G F K K S E E V S P R P A K T P L E P E E K K G F F E Q A K E R T P G F K K T E E V S P R P A

Secondary structure

SS confidence

Disorder: ?

Disorder confidence

. . . . 310 . . . . 320 . . . . 330 . . . . 340 . . . . 350 360

Sequence: K A A A H L T E T P L E P E E K K G F F D Q A K E R I P S H K K T E E V P P H P A Q S A S N E G A F S Q T E T P F E P E

Secondary structure

SS confidence

Disorder: ? — — ?

Disorder confidence

. . . . 370 . . . . 380 . . . . 390 . . . . 400 . . . . 410 420

Sequence: E K K G F L D K V K E K V P A H K T E E V P P P A E S A F S H T E T P F E P E E K K G F L D K V K E K E L A R K K T E E

Secondary structure

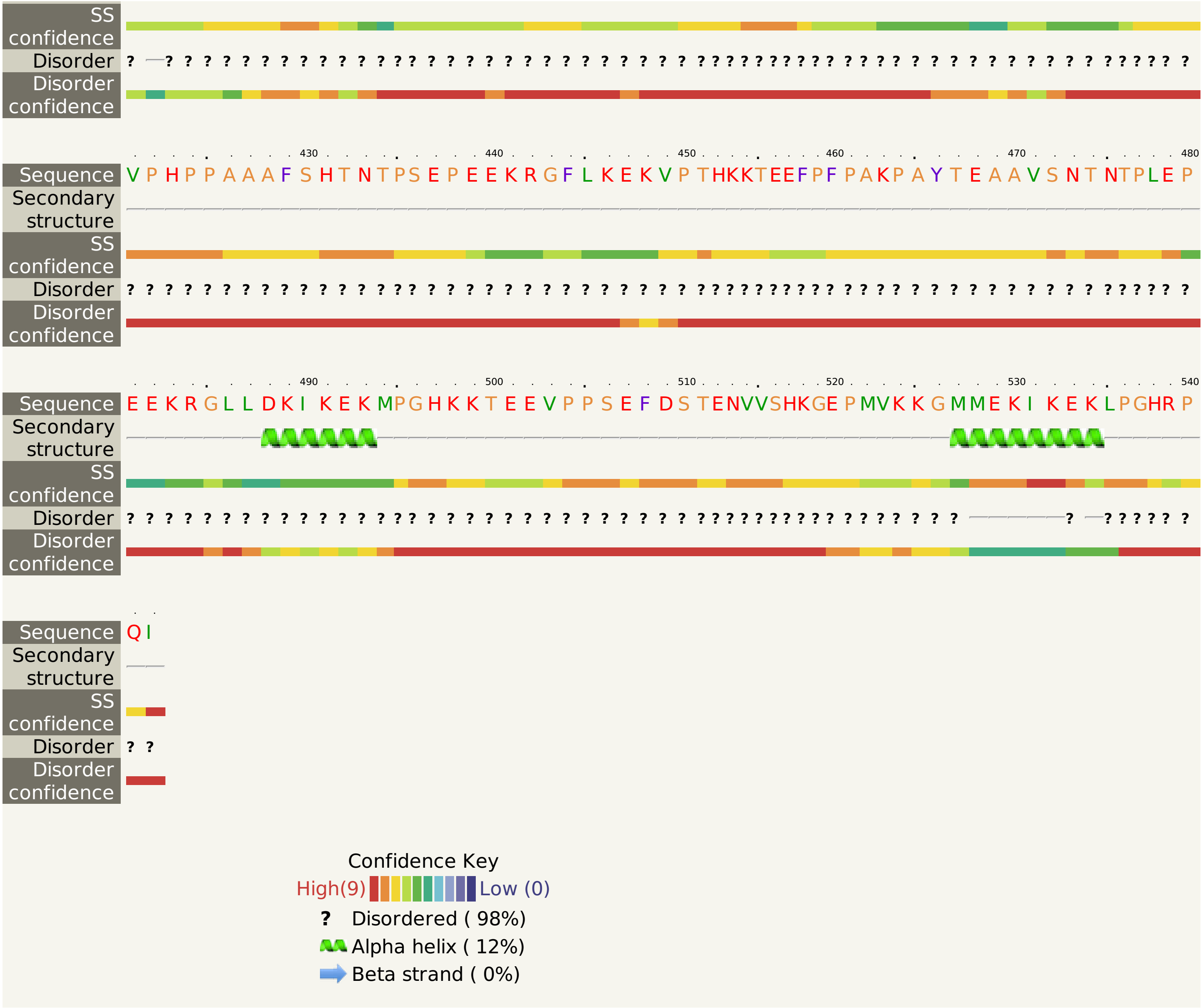

SS confidence
Disorder
Disorder confidence
430
440
450
460
470
480
Sequence VPHPPAAAFSHTNTPSEPEEKRGFLKEKVPTHKKTEEFPFPAKPAYTEAAVSNTNTPLEP
Secondary structure
SS confidence
Disorder
Disorder confidence
490
500
510
520
530
540
Sequence EEKRGLLDKIKEKMPGHKKTEEVPPSEFDSTENVVSHKGEPMVKKGMMEKIKEKLPGHRP
Secondary structure
SS confidence
Disorder
Disorder confidence
Sequence QI
Secondary structure
SS confidence
Disorder
Disorder confidence
Confidence Key
High(9)
Low (0)
Disordered (98%)
Alpha helix (12%)
Beta strand (0%)

Email	pedro.perdiguero@upm.es
Description	Populus_trichocarpa_Potri_003G138700
Date	Mon Sep 15 15:21:11 BST 2014
Unique Job ID	94e4a5d0930ef428

Secondary structure and disorder prediction

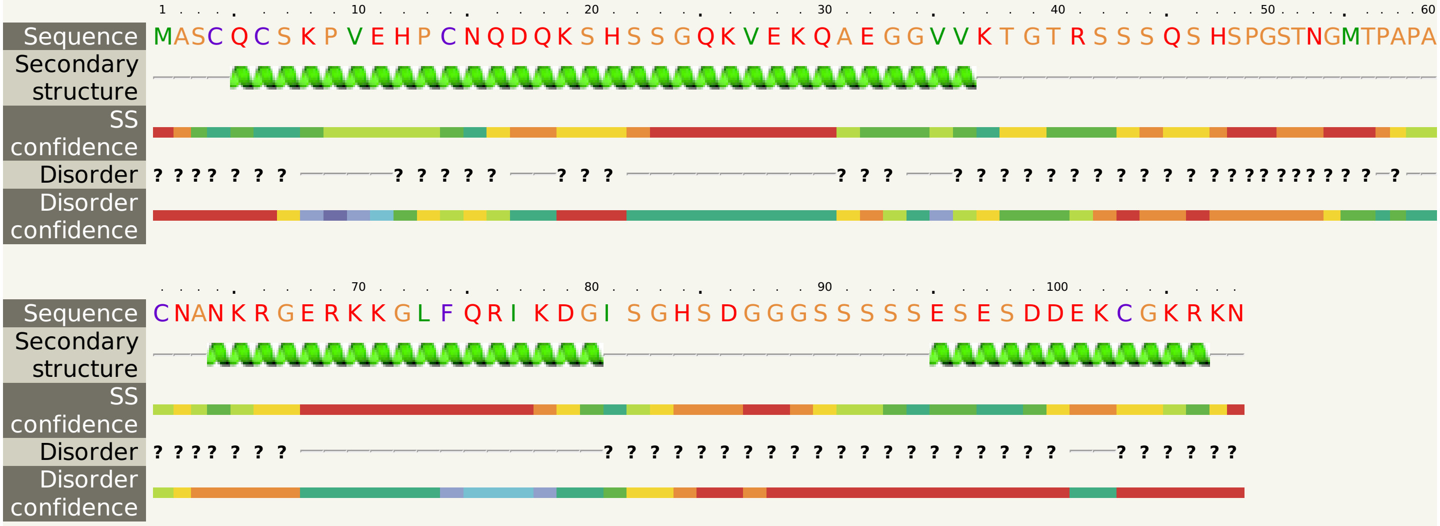

Confidence Key

High(9) Low (0)

? Disordered (68%)

Alpha helix (56%)

Beta strand (0%)

Email	pedro.perdiguero@upm.es
Description	Populus_trichocarpa_Potri_004G158500
Date	Mon Sep 15 15:19:03 BST 2014
Unique Job ID	c11d2d376445530e

Secondary structure and disorder prediction

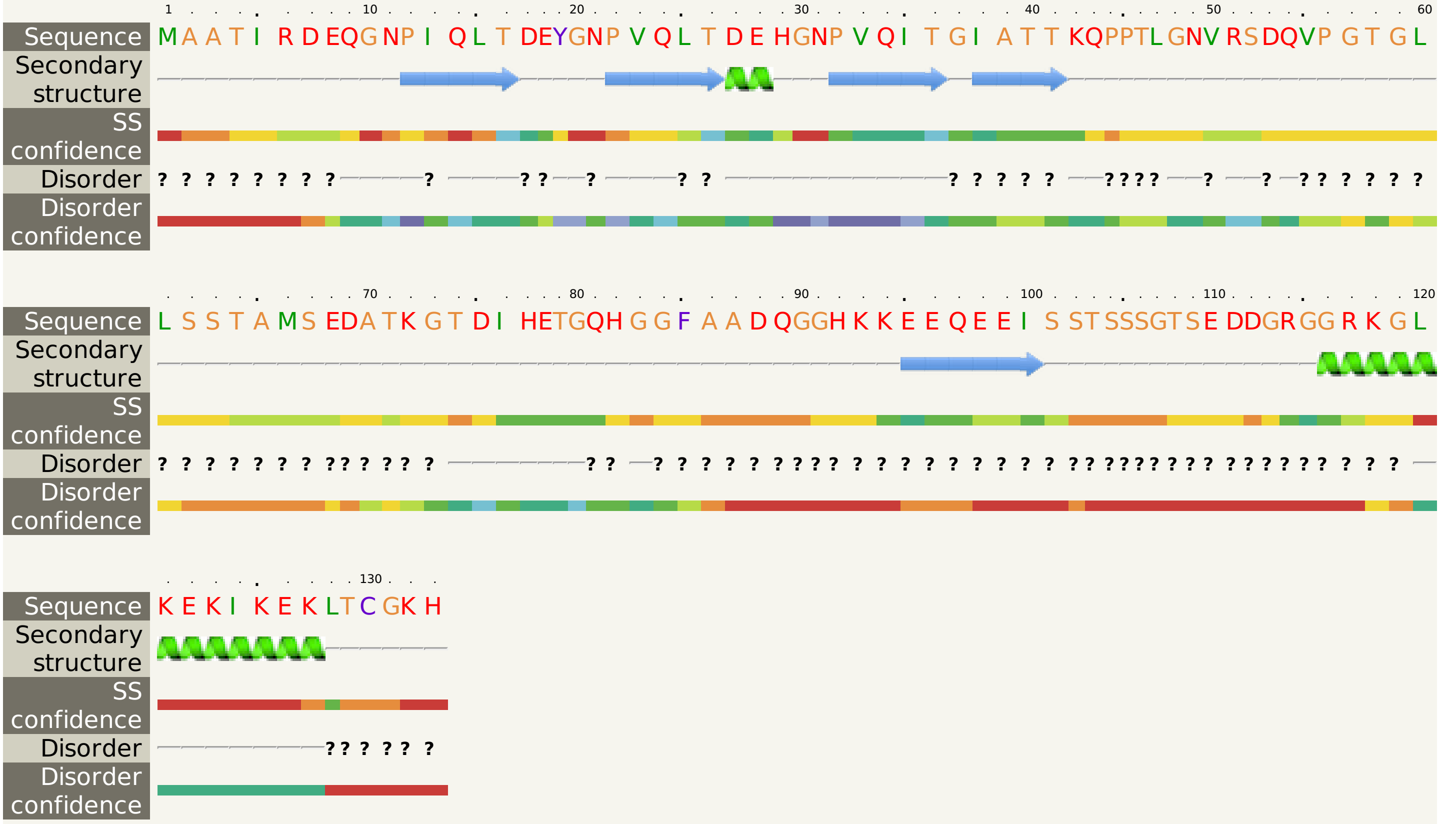

Confidence Key

High(9) Low (0)

? Disordered (66%)

Alpha helix (11%)

Beta strand (19%)

Email	pedro.perdiguero@upm.es
Description	Populus_trichocarpa_Potri_005G248100
Date	Mon Sep 15 15:19:24 BST 2014
Unique Job ID	90613f8de8f4be2d

Secondary structure and disorder prediction

Sequence (1–60): MAEENKSHEYETKVGEESGAVETKDRGLFDFLGKKEEEKPQEEVIGTEFEEKLQVSEPET

Sequence (61–120): KVEEEHKKKEEEEKKPTLFEKLHRSGSSSSSSSSDEEEGDDEEKKKKKKEKRSLKEKMKI

Sequence (121–180): SGEKGEEKEHEDTSVPVEVVHTETPHEPEDKKGFLDKIKEKLPGHKKADEVPPPAPEHVS

Sequence (181–225): PEAAVSHEGDAKEKKGLLEKIKEKLPGYHPKTEEEKEKEKESASQ

Confidence Key

High(9) … Low (0)

? Disordered (92%)

Alpha helix (21%)

Beta strand (0%)

Email	pedro.perdiguero@upm.es
Description	Populus_trichocarpa_Potri_009G120100
Date	Mon Sep 15 15:21:34 BST 2014
Unique Job ID	81484ffa67f02ddf

Secondary structure and disorder prediction

	1 . . . . 10 . . . . 20 . . . . 30 . . . . 40 . . . . 50 60
Sequence	MIAATIVDELGNPIQLTDEHGNPVKLTDEHGNPVHIAGVATTKQPPTLGDIISTDTVPGT
Secondary structure	
SS confidence	
Disorder	? ? ? ? ? ? ? — ? — ? — ? ? ? ? ? — ? — ? ? ? ? ? ? ? ? ? ? — ? ? ? ? ? ? ? ? ? ? ? ?
Disorder confidence	

	. . . . 70 . . . . 80 . . . . 90 . . . . 100 . . . . 110 120
Sequence	GHLSSTARSEDAMKGGLRETGHHGEVAGDQGGHKKEEHDETSSTSSSGSSEDDGQGGRRK
Secondary structure	
SS confidence	
Disorder	? ?
Disorder confidence	

	. . . . 130 . . . . 140 . . . . 150 . . . . 160 . . . . 170 180
Sequence	KKKGLKQKIKEKLTGGKHKEEHGHTVDVHTTTTGPAGEQYQEQEKKSVIEKIKGKLPGHH
Secondary structure	
SS confidence	
Disorder	? ? ? ? ? ? ? ? — ? — ? ? ? ? ? ? ? ? ? ? ? ? ? ? ? — ? ? ? ?
Disorder confidence	

	. . .
Sequence	SHH
Secondary structure	
SS confidence	
Disorder	? ? ?
Disorder confidence	

Confidence Key

High(9) Low (0)

? Disordered (80%)

Alpha helix (10%)

Beta strand (0%)

Phyre2

Email	pedro.perdiguero@upm.es
Description	Populus_trichocarpa_Potri_013G062100
Date	Mon Sep 15 15:20:10 BST 2014
Unique Job ID	dfd157030f4cbff5

Secondary structure and disorder prediction

Sequence: MAGIVHKIEDTLNNGGKKDERKGETQGGCNQQEHRGGAQGERKEGFVGQMKDKIPGGGGG (1–60)

Sequence: GVGGKGETQGGCNQQEHRGGAQGERKEGFVGQMKDKIPGGGGGGVGGVRGEGGEK (61–)

Rows: Sequence, Secondary structure, SS confidence, Disorder, Disorder confidence

Confidence Key

High(9) … Low (0)

? Disordered (77%)

Alpha helix (67%)

Beta strand (0%)

Email	pedro.perdiguero@upm.es
Description	Populus_trichocarpa_Potri_013G062200
Date	Mon Sep 15 15:19:45 BST 2014
Unique Job ID	c68cb71bafe4eaba

Secondary structure and disorder prediction

1 . . . . 10 . . . . 20 . . . . 30 . . . . 40 . . . . 50 60

Sequence: MAGIVHKIEDTLNIGGKKDERKGETQGGYNQQDHRGGAQGQRKEGFVGQMQDKILGGGGG

Secondary structure

SS confidence

Disorder: ? ? ? —————— ? —————— ? ? ? ? ? ? ?

Disorder confidence

. . . . 70 . . . . 80 . . . . 90 . . . . 100 . . . . 110 120

Sequence: GGGGMTQGGYNQQEHRGGAQGQRKEGFVGQMKDKIPGGGGGGVGGMTQGGYNQQEHRGGA

Secondary structure

SS confidence

Disorder: ?

Disorder confidence

. . . . 130 . . . . 140 . . . . 150 . . . . 160 . . . . 170 180

Sequence: QGQRKEGFVDKIKGKIPGGGGASGVGGMTQGGYNQQDHRGGAQVVRKEGFVDKIKGKIPG

Secondary structure

SS confidence

Disorder: ? ? ? ? ? — ? ———————— ? ——— ? ? ?

Disorder confidence

. . . . 190 . . . . 200 . . . . 210 . . . . 220 . . . . 230 . . . . . .

Sequence: GGGASGVGGMTQGGYNQQDHRGGAQGVRKEGFVDKIKGKIPGGGGASGVRGVGGEK

Secondary structure

SS confidence

Disorder: ? ———————— ? ? ? ? ? ? ? ? ? ? ? ? ? ? ? ? ? ? ?

Disorder confidence

Confidence Key

High(9) Low (0)

? Disordered (81%)

Alpha helix (30%)

Beta strand (0%)

Email	pedro.perdiguero@upm.es
Description	Populus_trichocarpa_Potri_013G062300
Date	Mon Sep 15 15:22:24 BST 2014
Unique Job ID	63fd8cf9bc3a13e0

Secondary structure and disorder prediction

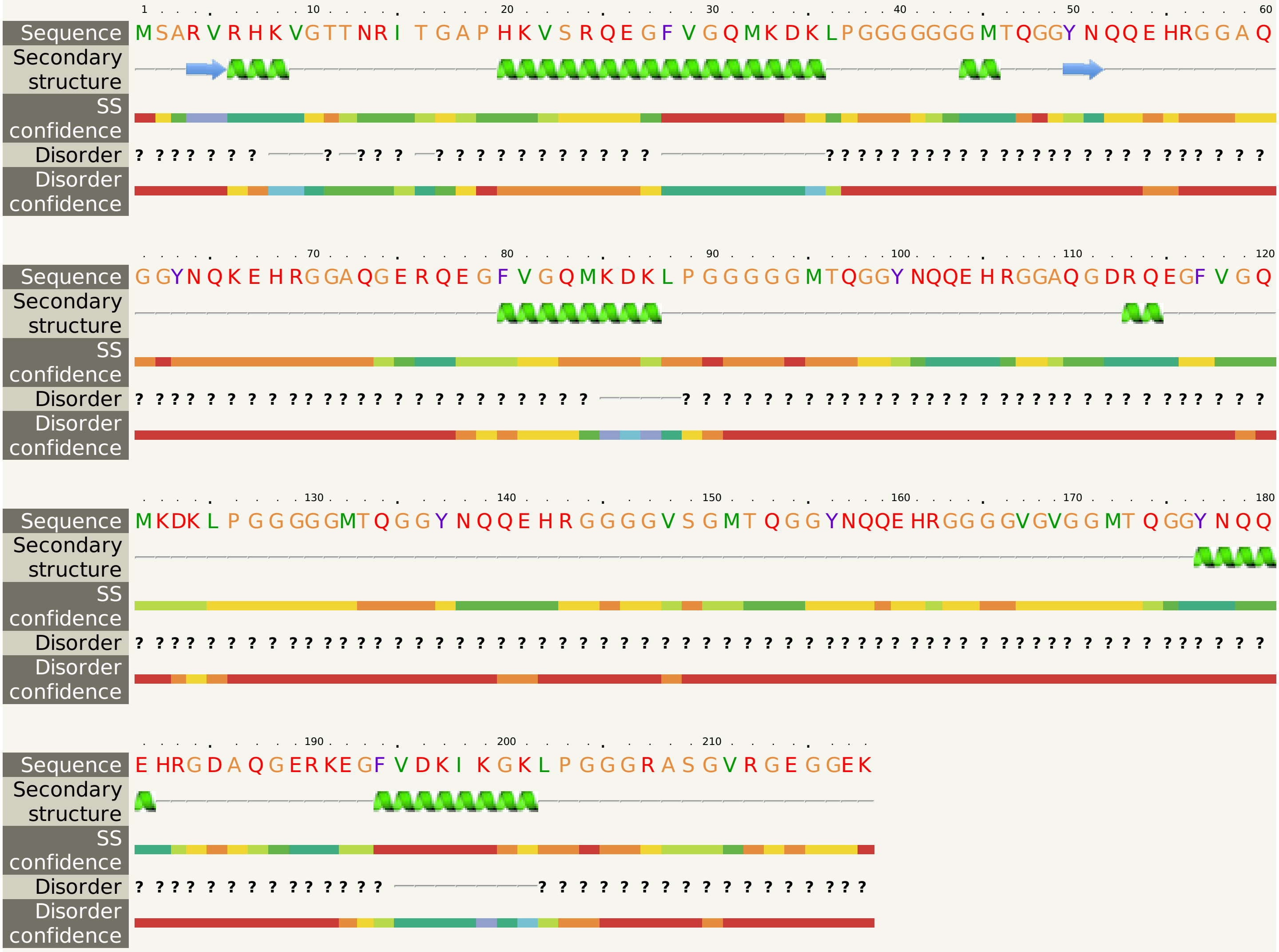

Confidence Key

High(9) … Low (0)

? Disordered (89%)

Alpha helix (20%)

Beta strand (2%)

Email	pedro.perdiguero@upm.es
Description	Populus_trichocarpa_Potri_013G062400
Date	Mon Sep 15 15:22:01 BST 2014
Unique Job ID	b9e5b4769e914abe

Secondary structure and disorder prediction

1 … 60

Sequence: M N E D L R D L F L I L N V A A R G S F H G A Y K C G T R F I H H F I S K A I S K R E K R R K K Y T M A G I M H K I E E

Secondary structure / SS confidence / Disorder / Disorder confidence

61 … 120

Sequence: T L N I G G K K D E R K G E T Q G G Y N Q Q D H R D G A Q G Q R K E G F V D Q M K D K I P G G G G G V H K G E T Q G G Y

Secondary structure / SS confidence / Disorder / Disorder confidence

121 … 180

Sequence: N H Q E Q G G Y N Q Q Q H R G G A Q G E R K E G F V D Q M K D K L P G G G G G M T Q G G Y N Q Q E H R G G A Q G G Y N Q

Secondary structure / SS confidence / Disorder / Disorder confidence

181 … 239

Sequence: Q E H R G D A Q G E R K E G F V D K I K G K I P G G G G G S G V R G E G G E K K K K K D K K K N E D G H S S S S D S D

Secondary structure / SS confidence / Disorder / Disorder confidence

Confidence Key

High(9) … Low (0)

? Disordered (72%)

Alpha helix (54%)

Beta strand (0%)
