# Supplementary material for: Novel dehydrins lacking complete K-segments in Pinaceae. The exception rather than the rule
Source: Front Plant Sci. 2014 Dec 2;5:682. doi: 10.3389/fpls.2014.00682 (PMC4251312; doi:10.3389/fpls.2014.00682)

Novel dehydrins lacking complete K-segments in Pinaceae. The exception rather than the rule

Pedro Perdiguero, Carmen Collada, Álvaro Soto

Suppl. Fig S3 exImage obtained in ConGenIE website (Nystedt et al., 2013) for the relative expression of MA_10427879g0010 from *Picea abies* (a dehydrin which also presents an incomplete and modified K-segment), based on RNA-seq data from different libraries.

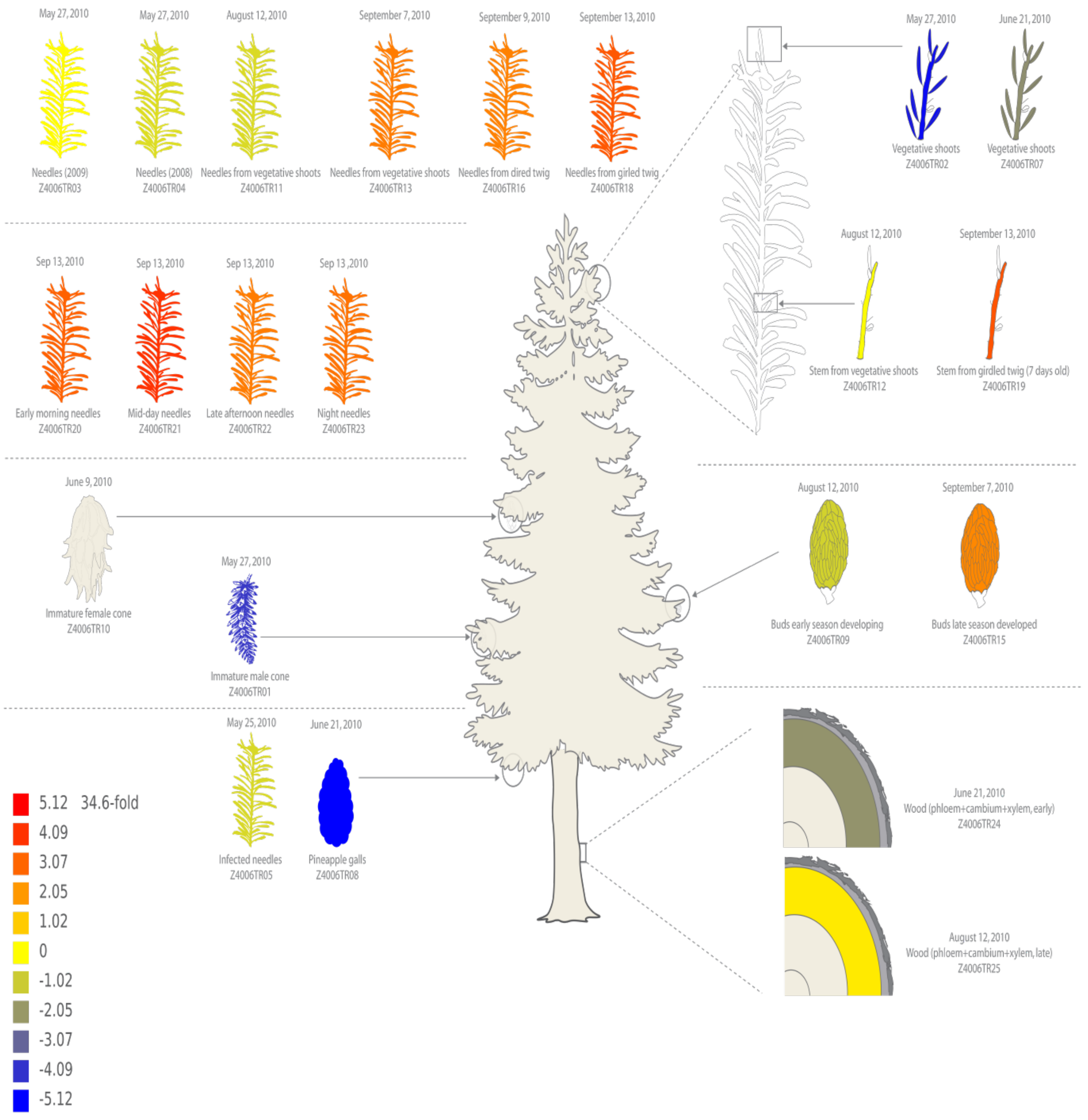

Supplement: Supplementary file 3 [file Image3.PDF]
